# Quantification of Diaphragm Mechanics in Pompe Disease Using Dynamic 3D MRI

**DOI:** 10.1371/journal.pone.0158912

**Published:** 2016-07-08

**Authors:** Katja Mogalle, Adria Perez-Rovira, Pierluigi Ciet, Stephan C. A. Wens, Pieter A. van Doorn, Harm A. W. M. Tiddens, Ans T. van der Ploeg, Marleen de Bruijne

**Affiliations:** 1 Biomedical Imaging Group Rotterdam, Departments of Medical Informatics & Radiology, Erasmus MC, Rotterdam, the Netherlands; 2 Department of Pediatric Pulmonology, Erasmus MC-Sophia Children’s Hospital, Rotterdam, the Netherlands; 3 Department of Radiology, Erasmus MC, Rotterdam, the Netherlands; 4 Department of Pediatrics, Respiratory Medicine and Allergology, Erasmus MC-Sophia, Rotterdam, the Netherlands; 5 Department of Neurology, Erasmus MC, Rotterdam, the Netherlands; 6 Centre for Lysosomal and Metabolic Diseases, Erasmus MC-Sophia, Rotterdam, the Netherlands; 7 Department of Pediatrics, Division of Metabolic Diseases and Genetics, Erasmus MC-Sophia, Rotterdam, the Netherlands; 8 Department of Computer Science, University of Copenhagen, Copenhagen, Denmark; University Children’s Hospital Bern, SWITZERLAND

## Abstract

**Background:**

Diaphragm weakness is the main reason for respiratory dysfunction in patients with Pompe disease, a progressive metabolic myopathy affecting respiratory and limb-girdle muscles. Since respiratory failure is the major cause of death among adult patients, early identification of respiratory muscle involvement is necessary to initiate treatment in time and possibly prevent irreversible damage. In this paper we investigate the suitability of dynamic MR imaging in combination with state-of-the-art image analysis methods to assess respiratory muscle weakness.

**Methods:**

The proposed methodology relies on image registration and lung surface extraction to quantify lung kinematics during breathing. This allows for the extraction of geometry and motion features of the lung that characterize the independent contribution of the diaphragm and the thoracic muscles to the respiratory cycle.

**Results:**

Results in 16 3D+t MRI scans (10 Pompe patients and 6 controls) of a slow expiratory maneuver show that kinematic analysis from dynamic 3D images reveals important additional information about diaphragm mechanics and respiratory muscle involvement when compared to conventional pulmonary function tests. Pompe patients with severely reduced pulmonary function showed severe diaphragm weakness presented by minimal motion of the diaphragm. In patients with moderately reduced pulmonary function, cranial displacement of posterior diaphragm parts was reduced and the diaphragm dome was oriented more horizontally at full inspiration compared to healthy controls.

**Conclusion:**

Dynamic 3D MRI provides data for analyzing the contribution of both diaphragm and thoracic muscles independently. The proposed image analysis method has the potential to detect less severe diaphragm weakness and could thus be used to determine the optimal start of treatment in adult patients with Pompe disease in prospect of increased treatment response.

## Introduction

Pompe disease (glycogen storage disease, type 2) is an inherited neuromuscular disorder characterized by progressive limb-girdle weakness and pulmonary insufficiency [1, 2]. Large variations in disease progression and manifestation are typical between adults with Pompe disease. For example, both age and limb-girdle weakness lack correlation with respiratory function [3]. However, disease duration and degree of respiratory muscle involvement are significant predictors of disease severity and rapid progression [4, 5]. Common consequences of diaphragm weakness are sleep-disordered breathing, ventilator dependency and respiratory failure, which is the major cause of death among patients [6, 7]. Enzyme replacement therapy (ERT), which was approved in 2006, has been demonstrated to positively alter the course of Pompe disease and stabilize or improve motor function [8], although pulmonary function may also continue to deteriorate [9]. Observational studies showed that ERT has a higher effect on patients with less severe symptoms and therefore suggest to start treatment early, before muscle damage becomes possibly irreversible [10, 11]. Quantitative methods for early and comprehensive diagnosis of diaphragm impairment are essential to determine the optimal start of ERT and to monitor and predict treatment response.

In current clinical practice, pulmonary function tests (PFT) are used to evaluate general respiration performance by breathing into a spirometer. In adult Pompe patients, early involvement of the diaphragm might stay undetected with PFT due to compensatory efforts of the intercostal, accessory and abdominal musculature [12, 13]. High resolution muscle magnetic resonance imaging (MRI) and computed tomography (CT) have been reported to be useful modalities to assess skeletal [14, 15] and respiratory muscle atrophy [16]. Yet, manual measuring of muscle thickness requires extensive anatomical knowledge and the structural analysis does not capture the actual functional effects on the respiratory system. In contrast, dynamic imaging enables direct functional analysis of the respiratory system. Lung and diaphragm motion have been previously investigated via x-ray fluoroscopy [17], ultrasonography [18–20], CT [21, 22] and MRI [23–27].

In dynamic imaging, multiple images (referred to as frames) acquired at different time points are combined to an image sequence. This can be achieved in 2D (e.g. at mid-sagittal plane in right and left lung or mid-coronal plane) [28–32], or in 3D [26, 33, 34].

The characterization of the respiratory system based on dynamic images can be categorized into two main types of features: geometry and motion measurements. Geometry features can be obtained from a single frame, independent of the other frames in the sequence. Typical geometry features described in literature [23, 24, 28, 30, 31, 35] are lung volume, lung size (in anterior-posterior, cranial-caudal and left-right direction), diaphragm length (2D images) or diaphragm surface area (3D images), both divided into zone of apposition and diaphragm dome. The diaphragm is a sheet of muscle that is attached to the ribs and separates the thoracic from the abdominal cavity. As a consequence of contraction and relaxation of the diaphragm during breathing, a part of this big muscle rests against the rib cage (referred to as zone of apposition) and the other part rests against the lungs and heart (referred to as diaphragm dome). Vostatek et al. [32] additionally investigated inclination, flatness and position of the diaphragm dome. The extraction of motion features requires tracking of landmarks through the whole image sequence. Multiple studies have investigated the excursion of the diaphragm during breathing in healthy subjects based on the established point correspondences [25, 29, 31].

To facilitate the extraction of geometry features, image segmentation methods are usually used to partition the image into meaningful segments. The segment boundaries (e.g. lung surface or diaphragm) can be by manually tracing object silhouettes [23, 24, 29, 31, 32], semi-automatically including little user interaction [34, 35], or fully automatically [27, 36]. Manual segmentation of the diaphragm and lungs is a very time-consuming and tedious task, especially in 3D images or images sequences. The automatic extraction of the diaphragm from radiological images is inherently difficult due to its small thickness (3.3 mm at full-expiration and mean thickening ratio of 1.8 at full-inspiration [37]). To the best of our knowledge, no methods have been reported to automatically segment the entire diaphragm (including zone of apposition) in either MR or CT images. In high-resolution CT images, the diaphragmatic dome has been segmented by fitting a quadratic surface [38] or a thin-plate model [39] to the most inferior voxels of a lung segmentation. Automated lung segmentation in static 3D MRI data has been proven feasible, e.g. by using 3D region growing [36], confidence-connectedness segmentation [26] or statistical shape models in combination with deformable mesh segmentation [40]. In dynamic MRI scans, the automatic delineation of the diaphragm is more challenging due to the poor quality of scans (i.e. low spatial image resolution, low signal-to-noise ratio, artifacts at tissue borders) resulting from the high demands on temporal resolution.

Motion features have previously been extracted in two different ways: segmentation- and registration-based. Plathow et al. [27] applied deformable mesh segmentation successively to the 3D frames of an image sequence while incorporating temporal coherence. Yet, the robustness of this approach with respect to image artifacts (e.g. ghosting at lung tissue borders) was not addressed and spatial/temporal accuracy and smoothness of the segmentation were not evaluated. Lung motion has also been represented in terms of deformation fields which are estimated using intensity-based non-rigid image registration [22]. Recent advances in this field aimed at decreasing computation time [34, 41] or incorporating temporal regularization to enforce smooth lung motion [42].

In this paper, we present an automated method to quantify the contribution of the different muscles involved in the respiratory system in order to get deeper insights into the diaphragm impairment of adult Pompe patients compared to healthy controls. Dynamic 3D MRI is used to capture a slow full exhalation breathing maneuver. We apply a registration-based approach [42] to obtain deformation fields describing the motion between frames of the image sequence. Lung segmentations are propagated from a reference frame to all other frames, thus enabling us to quantify and investigate the movement of the diaphragm and chest wall during the respiratory maneuvers. The main contributions of this paper are 1) the estimation of lung motion achieved through modification of an inhomogeneity correction and an image registration method, 2) the automatic extraction of lung surface regions adjacent to the diaphragm and chest wall based on lung segmentations, 3) the automatic computation of geometry and motion features for different lung surface parts and 4) the evaluation of the relationship between the proposed image features and PFT features as well as their ability to discriminate between patients with Pompe disease and healthy controls.

## Materials and Methods

### Ethics statement

Written informed consent was obtained from all participants. The study protocol was approved by the Medical Ethics Committee at the Erasmus MC University Medical Center Rotterdam (Amendment 7 to protocol MEC-2007-103).

### Data acquisition

The presented study includes 16 adults, of which 10 were diagnosed with late-onset Pompe disease (referred to as *P01*—*P10*) and 6 age- and gender-matched controls (referred to as *C01*—*C06*). At data acquisition, the median age of the patients was 46 years (range: 32–66) and for controls 43 years (range: 27–55). Both groups consisted of 50% females. Three patients required nocturnal ventilation and one was wheelchair dependent. The median time since diagnosis was 16 (range: 9–30) years and all patients received enzyme replacement therapy (range: 0–7 years, median duration: 5.5 years).

All subjects were scanned on a 3T GE Signa 750 MRI scanner (General Electric Healthcare, Milwaukee, USA) using a 32-channel torso coil. The exact parameters of the acquisition procedure are described in [43] and in the table of S1 Table. Static, full-inspiration and full-expiration scans were acquired during 12-seconds breath-hold using a 3D RF-spoiled gradient echo sequence (sagittal volume acquisition, 3 mm slice thickness, 3.6 × 2.8 × 3.0 mm acquired resolution, approx. 1.4 × 1.4 × 1.5 mm interpolated voxel size). A dynamic sequence was also acquired while the subjects performed a slow exhalation maneuver from total lung capacity (TLC) to residual volume (RV). This sequence contains 48 3D volumes which were continuously acquired at a rate of approximately 2.5 volumes per second. Fast acquisition was achieved using the time resolved imaging of contrast kinetics (TRICKS) technique [44] and a reduced spatial resolution (sagittal volume acquisition, 12 mm slice thickness, 4.8 × 6.0 × 12.0 mm acquired resolution, approx. 1.9 × 1.9 × 6 mm interpolated voxel size). To guarantee the correct execution of the maneuver during static and dynamic imaging, both the preliminary training and the scanning were controlled with an MR-compatible spirometer and instructions were given by a certified lung function technician who also supervised the quality of the maneuver. An example of the acquired sequence for one Patient and one control are provided in the videos in S1 and S2 Videos.

The dynamic sequences were manually cropped, discarding the tidal breathing after full exhalation. The sequence length *T* (expressed as mean±std) for all subjects thus decreased to 36 ± 8.9 frames (patients: 33.6 ± 9.8, volunteers: 40 ± 5.3).

A trained medical observer manually segmented right and left lung in both static scans (inspiration and expiration) of each subject by tracing the lung surface in every second axial slice using 3D slicer (www.slicer.org). Slices without annotations were filled automatically via interpolation.

Pulmonary function tests (PFT) were also performed prior to the MRI scan to measure vital capacity (VC), forced vital capacity (FVC), forced expiratory volume in one second (FEV1), peak expiratory flow (PEF) and maximum inspiratory and expiratory pressure (MIP, MEP). The measurements VC, FVC, FEV1 and PEF were acquired both in sitting and supine position and expressed as absolute values as well as percentage with respect to predicted values. The postural drop ΔFVC was measured as the relative difference between FVC in sitting and supine position. A drop of >25% is commonly considered an indicator of diaphragm weakness [12, 13].

### Methods overview

In order to automatically extract characteristics of respiratory muscle dynamics, the motion of the lungs and surrounding structures was captured via non-rigid image registration of the dynamic image sequence resulting in a dense deformation field. Based on these deformation fields, the contribution of the different muscular groups involved in the respiratory maneuver is derived. By tracking the bottom of the lung surface the diaphragm motion can be investigated in detail, while the chest wall contribution is inferred by evaluating the movement of the anterior-posterior and left-right surfaces of the lung.

**Fig 1:**
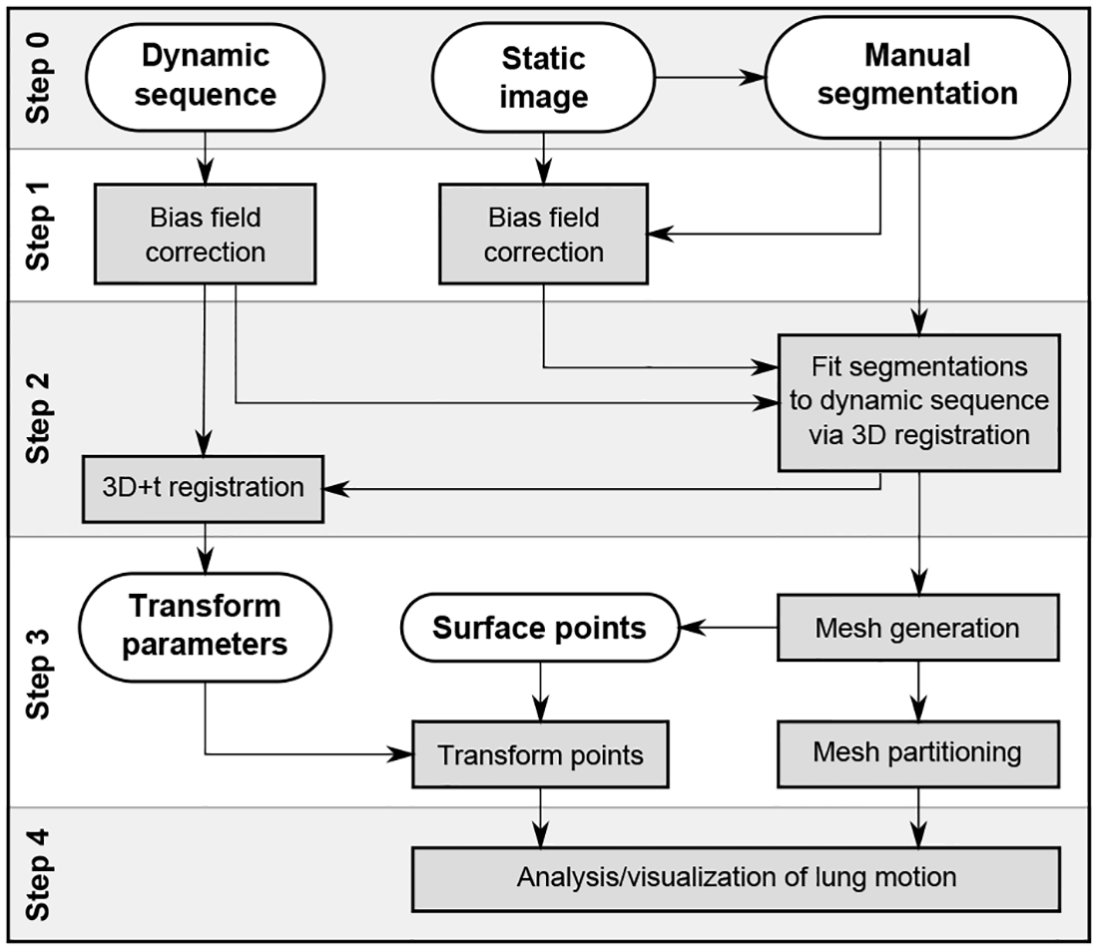
Overview of the image analysis pipeline. The gray boxes present the general steps necessary to process the input images (dynamic sequence, static image and manual segmentation of the static image) and to derive motion and geometry features of the diaphragm and lung surface.

Fig 1 shows a schematic overview of the proposed image processing pipeline. The main framework can be divided into four steps: preprocessing, deformation field estimation, lung surface partitioning, and motion analysis. The bash scripts for the first two steps as well as the MATLAB source code of the latter two steps are available upon request at marleen.debruijne@erasmusmc.nl. In the following, all steps are presented in more detail.

### Image preprocessing

The MRI images acquired in this study are affected by severe intensity inhomogeneity mostly due to the usage of surface coils (see video in S2 Video). Since motion estimation is performed via an intensity based registration approach in this framework, it is crucial to correct for this MRI artifact in order to correctly compute the motion of the diaphragm. Our framework utilizes the method N4ITK [45], an extension of the well-known inhomogeneity correction algorithm N3. The parameters of N4ITK, especially the spline distance, where finely tuned to obtain optimal results for the presented MRI acquisition setup. The parameter optimization resulted in the choice of two fitting levels (max. 500 iterations each) and an initial spline distance, i.e. control point spacing, of 100 mm in spatial domain. For the correction of the dynamic scans two control points were used in the time dimension. The initial results could further be considerably improved by developing an iterative approach to generate masks that define the image regions from which the bias field is estimated. The mask includes the body but excludes all air-filled regions, specifically the lungs and the image background. To generate such masks, the image is thresholded using Otsu’s method [46] and the intensity bias field is computed using only the bright regions. The resulting image with reduced intensity inhomogeneity is then thresholded again to obtain a new, more accurate, mask. In the next iteration this new mask is used to correct the original image with the N4ITK algorithm. Three iterations were used, as visual inspection showed that more than three iterations produced no further improvement, and the resulting images were of sufficient quality for the next image processing steps. During bias field correction of the static scans, the corresponding manually generated lung mask was subtracted from the mask obtained via Otsu’s method.

### Motion estimation

The motion of the lungs in a dynamic sequence was estimated via 3D+t groupwise non-rigid registration as described by Metz et al. [42] using Elastix 4.7 [47]. The underlying deformation model uses a four-dimensional free-form B-spline that provides smoothness in the three spatial dimensions and in the time dimension. All 3D volumes of a sequence are aligned in an implicitly defined mean reference frame by minimizing the variance of intensity values over time. Registration was achieved using an adaptive gradient descent optimizer and a multi-resolution scheme with four resolution levels. Besides the B-spline grid spacing of the estimated transformation, all default values of the method described in [42] were used. The exact values of the chosen grid configuration are presented in the results section and the parameter files utilized by Elastix are provided in S1 File.

In order to restrict the registration to use image information in and around the lung, a lung mask was created in the following manner: The manual segmentations of the static full-inspiration and full-expiration scans, $M_{in}^{s}$ and $M_{ex}^{s}$ (with *s* referring to the static scans), were non-rigidly registered onto the first and last frames of the dynamic sequence respectively. Mutual information and a 4-level coarse-to-fine pyramid approach with a final B-spline spacing of 50 × 50 × 50 mm was used, resulting in the masks $M_{1}^{d}$ and $M_{T}^{d}$ (with *d* referring to the dynamic scans, 1 denoting the first and *T* the last frame). The union of the two binary masks was then dilated by 50 mm.

The above explained 3D+t non-rigid registration of the dynamic sequence estimates the B-spline coefficient vectors of the so-called *forward* (B-spline) transformation, which transforms each 3D volume to a mean reference frame. Additionally, an *inverse* transformation has to be computed to transform volumes of the mean reference frame to any time point in the original coordinate system (see [42]). When both *forward* and *inverse* transformation are concatenated, a deformation field can be established which can be used to transform points or images of one specific time point, for instance the last frame (full-expiration), to any other time point.

### Lung surface partitioning

All the hereafter presented methods are implemented in MATLAB (R2013b, MathWorks, Inc.). A lung surface mesh, consisting of a set of vertices *V* and a set of triangles *F*, is obtained from the voxel-based full-expiration lung segmentation $M_{T}^{d}$, by utilizing the CGAL 3.5 3D mesher (based on Delaunay refinement) which is accessed through the mesh processing toolbox *iso2mesh* [48]. The vertices of this 3D triangle mesh were propagated from the last time point to all other time points by applying the previously computed dense deformation field. This procedure results in a 3D+t lung segmentation in which points on the lung surface are directly traceable throughout the breathing maneuver. For further processing, outward-pointing normals are computed for each triangle. An example for such lung meshes is provided in the files of S1 Data.

**Fig 2:**
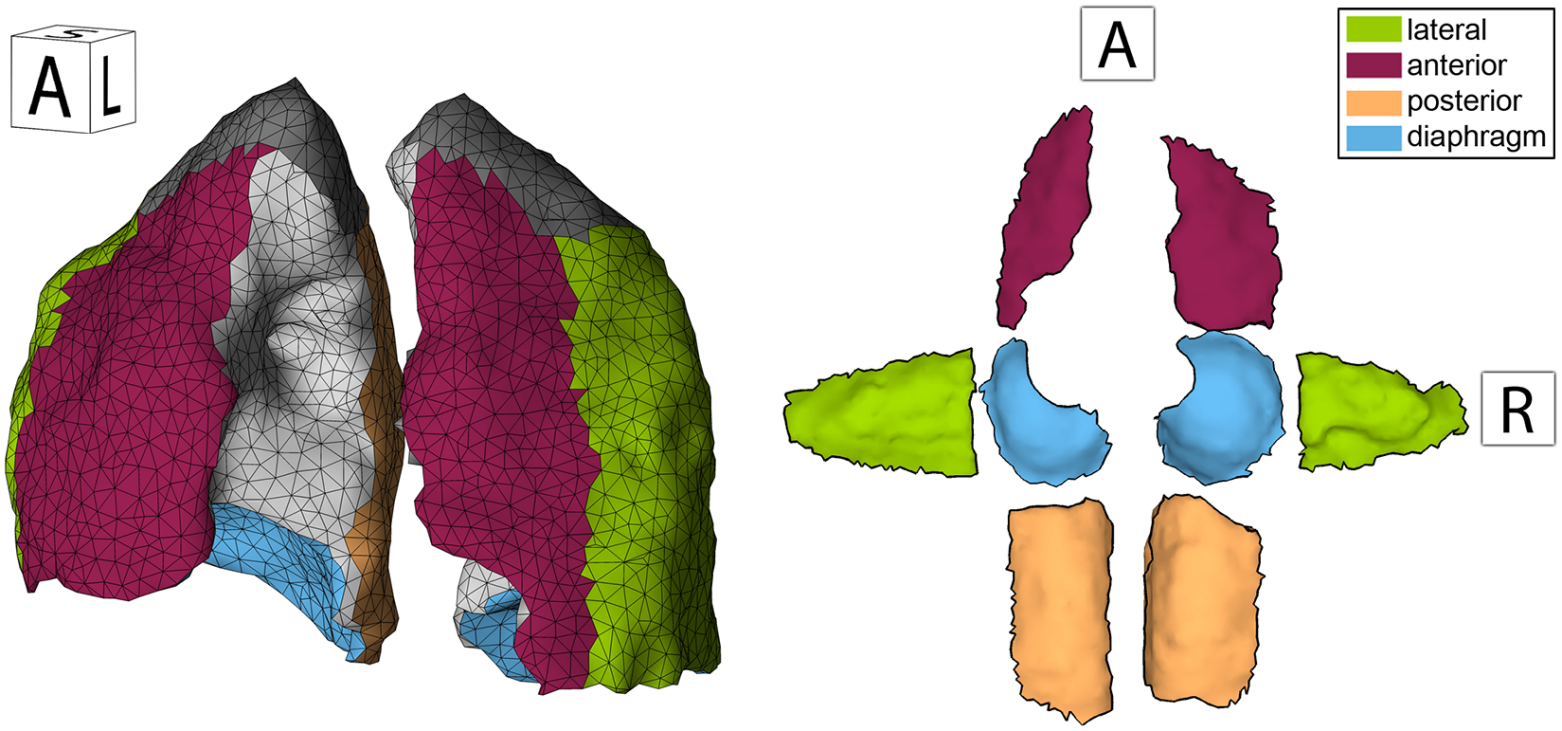
Mesh representation of 3D lung segmentation. A 3D mesh of the lungs is presented in the left image. The surface is colored to distinguish between the different surface segments. On the right, the same mesh is shown after unfolding the individual surface segments into a plane. The orientation labels indicate the viewing direction.

**Fig 3:**
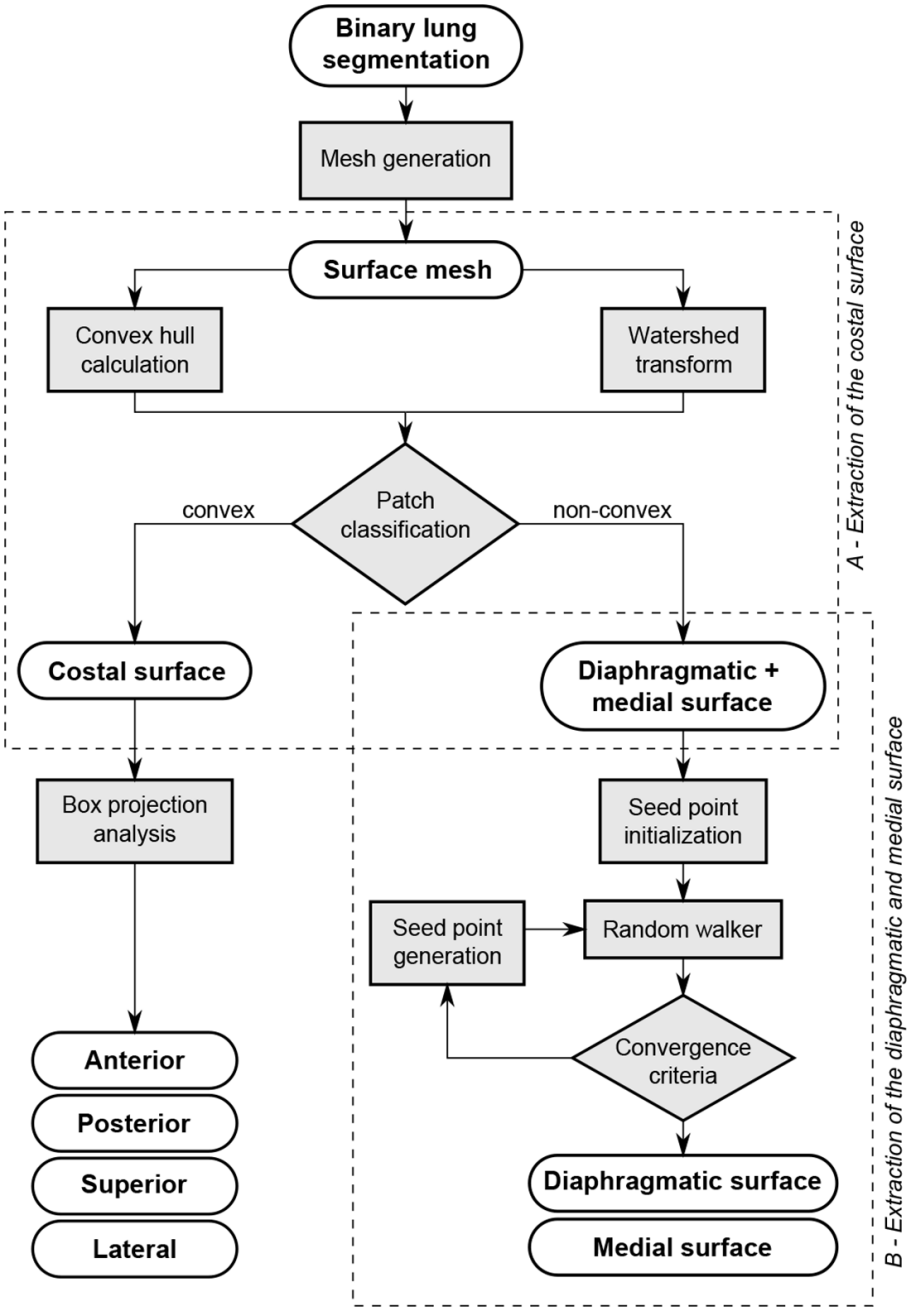
Overview of the lung surface partitioning procedure. The diagram shows the individual steps to subdivide the surface mesh of a binary lung segmentation into six parts. In the two main parts the costal surface is extracted and the residual surface is divided into diaphragmatic and medial surface.

The main aim of our study was to assess the contribution of the different respiratory muscles during breathing. Therefore, chest wall and diaphragm motion were investigated separately by analyzing the movement of lung surface regions adjacent to the respective anatomical structures. To this end, the lung surface mesh at full exhalation was subdivided into three major subsurfaces (Fig 2, left) associated with the chest wall (anterior, posterior, lateral, and superior parts), mediastinum (medial parts adjacent to the heart), and diaphragm (inferior part). This subdivision is achieved, for each individual lung, in the two steps illustrated in Fig 3 and detailed as follows.

#### Extraction of the costal surface

The extraction of the lung surface region adjacent to the chest wall, called costal surface, is based on the main anatomical observation that it is the only large surface area which is convex. Both the diaphragmatic and medial lung surface regions are non-convex due to the adjacent organs (heart and liver respectively). A watershed algorithm [49] is applied to the whole mesh in order to generate a subdivision into patches (watershed basins), where each patch has a relatively homogeneous curvature and nodes with high curvature (i.e. surface ridges) only lie on the boundary between patches. The maximal principal curvature of the surface, which is computed at each vertex of the mesh, serves as base feature for the watershed algorithm. The costal surface is extracted by merging the patches which have a small distance (at least one quarter of its triangles closer than 10 mm) to the convex hull of the lung.

For visualization purposes the costal surface is further subdivided into anterior, posterior, superior and lateral parts, based on which of the six faces (anterior, posterior, lateral, medial, superior, inferior) of the lung’s bounding box are intersected by the triangles normal vectors.

#### Extraction of the diaphragmatic and medial surface

Due to the high inter-subject variability of lung and heart shape and size, the differentiation between medial and diaphragmatic lung surface is inherently difficult. A flexible and robust approach is the random walker strategy which was first described by Leo Grady [50] for interactive medical image segmentation. The random walker operates on arbitrary graphs consisting of a set of nodes *P* and a set of edges *E*. Given seed nodes for each target region, it outputs a probability vector for each non-seed node which expresses the probability that the node belongs to any of the specified target regions. The following four steps describe in detail how the random walker is applied to separate the medial from the diaphragmatic surface.

**(1) Graph definition**—A graph is constructed from the lung surface mesh, excluding the previously determined costal surface. Mesh triangle center points are used as graph nodes (**p**_*i*_ ∈ *P*) and graph edges (*e*_*ij*_ ∈ *E*) are established between adjacent triangles. After assigning labels to the graph nodes based on the presented procedure, node labels are transferred back to the corresponding triangles of the original mesh.

**(2) Edge weight computation**—Each edge *e*_*ij*_ of the graph is assigned a weight *w*_*ij*_ between 0 and 1 expressing the probability that node **p**_*i*_ and **p**_*j*_ belong to the same target region. The applied weight function $w_{ij}=\exp\left(-d_{ij}^{2}\right)$ uses a distance measurement *d*_*ij*_ which is adopted from Zhang et al. [51]. It takes the euclidean distance between the nodes **p**_*i*_ and **p**_*j*_, the deviation of their surface normals **n**_*i*_ and **n**_*j*_, and the normal curvature *κ*_**p**_*i*_**p**_*j*__ into account:

$$\begin{array}{c} d_{ij}=w_{1}\left|\right|p_{i}-p_{j}\left|\right|+w_{2}\left|\right|n_{i}-n_{j}\left|\right|+w_{3}f\left(\kappa_{p_{i}p_{j}}\right) \end{array}$$ (1)

The coefficients *w*_1_, *w*_2_ and *w*_3_ are set according to [51]. Contrary to the adopted method where concave edges are considered relevant partition boundaries, in our case convex edges need to be emphasized:

$$\begin{array}{c} f\left(\kappa \right)=\{\begin{array}{cc} 5\kappa , & \kappa \geq 0\\ |\kappa |, & \kappa <0 \end{array}\;\;\;\text{with}\;\text{normal}\;\text{curvature}\;\kappa \end{array}$$ (2)

**(3) Initial seed node selection**—The initial seed node selection is based on anatomical knowledge and the previously defined mesh patches from the watershed transform (see previous section “Extraction of the costal surface”). After patch generation, patches are selected which can be assigned to one of the two target regions with high certainty. The most posterior patch can safely be assigned to the label *diaphragmatic surface*. The most superior patch and all patches which are at least partially occluded when viewed from a point centrally located below the lung are assigned to the *medial surface*. To further decrease the number of patches with an unknown membership, an unlabeled patch acquires the label of an adjacent patch if their normals diverge less than 30°and if no high ridge separates the patches into different regions (based on average weight $\bar{w_{ij}}$ between nodes of both patches). This procedure is repeated until no more patches can be labeled. In 15 out of 32 lung meshes, all patches were labeled, rendering the succeeding random walker step redundant. At last, a single seed node is obtained from every labeled patch by determining the graph node which is associated with the central triangle of the respective patch.

**(4) Iterative random walker surface partitioning**—Based on the edge weights defined in step (2) and initial seed nodes from step (3), the random walker algorithm is executed and the resulting probability maps are thresholded with high values (*p*_*diaphragm*_ > 0.8 and *p*_*medial*_ > 0.6) in order to derive a first partitioning of the diaphragmatic and medial surface. In the next iteration, seeds are randomly placed in the last-obtained partitions and added to the initial set of seed nodes. The iteration process is terminated when the partitions no longer change significantly (number of triangles is partition changed less than 3% to previous partition). Ultimately, the final partitioning results from assigning nodes with *p*_*diaphragm*_ > 0.8 to the diaphragmatic surface and nodes with *p*_*diaphragm*_ ≤ 0.8 to the medial surface.

### Motion analysis and visualization

Using the deformation field and the partitioned surface of the lung, we extracted the following features to assess the motion of different respiratory muscles and highlight the diaphragmatic weakening caused by Pompe disease. All features listed below are extracted from both left and right lung separately.

#### Basic features

For each time point *t* ∈ [1..*T*] the volume $V(M_{t}^{d})$ is computed from the lung mesh by using the method proposed in [52]. In addition to the time-dependent volume measurement, image-derived values for inspiratory volume ($V(M_{1}^{d})$), residual volume (**B1**: $V(M_{T}^{d})$, also referred to as expiratory volume) and vital capacity (**B2**: $V\left(M_{1}^{d}\right)-V\left(M_{T}^{d}\right)$) were computed. Furthermore, the ratio between total lung capacity and residual volume (**B3**: $V\left(M_{1}^{d}\right)/V\left(M_{T}^{d}\right)$) was determined.

As a coarse estimate of independent muscle contribution, we determined the change of lung size separately in the craniocaudal (CC) direction, which is mostly driven by the diaphragm, and in the anteroposterior (AP) direction, which is mostly determined by motion of the chest wall. The CC size is defined by the vertical distance between the most superior point of the whole lung (lung apex) and the most superior point of the diaphragmatic surface (diaphragm apex). The AP size is defined as the distance between the most anterior point in the lung surface to the most posterior point in the AP direction. Time-dependent size measurements are derived by computing the CC and AP size of the lungs at each time point *t* ∈ [1..*T*]. Furthermore, the increase in CC and AP size is computed (**B4**: $CC\left(M_{1}^{d}\right)/CC\left(M_{T}^{d}\right)$ and **B5**: $AP\left(M_{1}^{d}\right)/AP\left(M_{T}^{d}\right)$) and the ratio between these two features (**B6**: **B4**/**B5**) is also reported.

#### Advanced features

Diaphragm and chest wall contribution can be measured by directly computing the volume displaced by the diaphragmatic surface and the costal surface. At first, the set of triangles *D* ⊂ *F* and *C* ⊂ *F*, representing the diaphragmatic and costal surface respectively, are eroded by two triangle strips to eliminate unreliable measurements at the edges. The volume displaced by a single triangle f (*f* = △**v**_1_ **v**_2_ **v**_3_ ∈ *F* and **v**_*i*_ ∈ *V*) is obtained by computing the volume of the polyhedron that connects the vertices of the triangle at time point *t* with the vertices of the same triangle at time point *t* + 1. The polyhedron volume can be negative or positive depending on whether the triangle moves in the direction of its normal vector or not. The volume displaced by the whole diaphragmatic surface between time points *t* and *t* + 1, denoted as $\Delta V_{D}^{t}$, is computed by accumulating the polyhedron volumes of all triangles in *D*:

$$\begin{array}{c} \Delta V_{D}^{t}=\sum_{f\in D}V\left(v_{1}^{t}v_{2}^{t}v_{3}^{t}v_{1}^{t+1}v_{2}^{t+1}v_{3}^{t+1}\right) \end{array}$$ (3)

At last, the volume which is displaced during the entire breathing maneuver is obtained by summing the displaced volumes $\Delta V_{D}^{t}$ over all time points.

$$\begin{array}{c} \text{A1:}\;\Delta V_{D}=\sum_{t=1}^{T-1}\Delta V_{D}^{t} \end{array}$$ (4)

The volume displaced by the costal surface, $\Delta V_{C}^{t}$ and Δ*V*_*C*_ (**A2**), is computed in an analogous manner. Note that for inter-subject comparison, the features **A1** and **A2** are normalized based on the residual volume in order to compensate for differences between subjects (e.g. related to age, height and gender). Furthermore, we define the contribution of the diaphragm as:

$$\begin{array}{c} \text{A3:}\;\frac{\Delta V_{D}}{\Delta V_{C}+\Delta V_{D}} \end{array}$$ (5)

In literature it has been reported that in healthy subjects the cranial excursion of the diaphragm is posteriorly greater than anteriorly. This especially applies to the right hemidiaphragm (half of the diaphragm) [53]. Excursion variations within the diaphragm have been observed with various imaging modalities, such as ultrasonography [18], fluoroscopy [53] and MRI [23, 28]. In order to quantify anterior-posterior kinematic variations in our data, the mesh triangles of the diaphragmatic surface *D* were divided into two groups based on their position at full-expiration. The mid-coronal plane of the lung bounding box was utilized as a threshold. Consequently, the maximum CC displacement of each triangle was calculated with respect to the full-inspiration state and a weighted average was computed for the anterior and posterior part separately, taking the triangle area into account. The ratio between mean anterior and posterior diaphragm displacement was calculated for the diaphragmatic surface of the right lung (**A5**). The left lung was discarded from this analysis since a large portion of the left hemidiaphragm was missing due to the heart position. Additionally, the mean displacement of the whole diaphragmatic surface was reported (**A4**).

Furthermore, we determined the orientation of the right diaphragm dome as a surrogate measurement of the whole diaphragm shape. At each time point, a weighted average of triangle orientation (angle between normal vector and CC axis) is computed in the sagittal plane, using the triangle area as weights. Diaphragm orientation at full-inspiration (**A6**) and the largest signed difference between the orientation at the start of motion and any time point between start and end of motion (**A7**) were also obtained. The start and end of motion were defined as the time points at which 10% and 90% of the volume change was reached, respectively.

#### Visualization

A visual representation was developed to show the magnitude of displacement over the whole lung surface. To generate a 2D representation, the diaphragmatic surface and the anterior, posterior, left lateral and right lateral parts of the costal surface were associated with the bottom, front, back, left and right side of a rectangular box respectively. Subsequently, the box was unfolded by rotating its sides into the bottom plane resulting in a 2D map which gives an overview of the entire lung surface (Fig 2, right). The maximum excursion of each point along one of the three major axes (LR, AP, CC) is visualized in color on the 2D surface maps. Thus, the eight displayed lung surface parts show the following type of movement:

- lateral lung surface parts: maximum excursion along LR axis (positive towards mid-sagittal plane),
- anterior/posterior lung surface parts: maximum excursion along AP axis (positive towards mid-coronal plane),
- diaphragm parts: maximum excursion along CC axis (positive in cranial direction).

## Results

### Motion estimation

#### Choice of registration parameters

For the B-spline based 3D+t *forward* transformation isotropic B-spline grid resolutions of 10, 20, 40 and 80 mm in spatial domain and a spacing of 1, 2, 3 and 5 frames in time domain were evaluated. The computed transformations were used to transform the manual segmentations $M_{1}^{d}$ and $M_{T}^{d}$ of the first and last frame (respectively) to the mean reference frame. For every subject the Dice overlap scoring between both masks was computed and mean values and standard deviations were calculated for the whole population. A perfect overlap of both masks would result in a Dice score of 1. The results show that the best spatial resolution is 20 × 20 × 20 mm which gives a mean Dice overlap of 0.87 ± 0.015. However, temporal spacing has very little effect on the deformation fields of the first and last frame (the only time-points with manual segmentations) and could not be optimized in this fashion. Visual inspection of the registration results showed that with a very large temporal spacing (e.g. 5 frames) the start and end of the main respiratory motion are not captured correctly. On the other hand, temporal smoothing is necessary to compensate for ghosting artifacts. A temporal spacing of 2 frames was chosen as a trade-off between accuracy and ghosting compensation.

#### Registration evaluation via manual measurements

The presented method for lung motion estimation was evaluated quantitatively by comparing AP and CC lung size measurements based on the 3D+t lung meshes and landmarks extracted manually from the dynamic image sequence by two observers (Mogalle and Perez-Rovira). One observer selected the axial slice in the middle between lung apex and highest point of the whole diaphragm at full-expiration. The maximal AP lung size was then assessed by both observers using this predefined subject-specific slice. The CC lung size was measured from the lung apex to the diaphragm apex. For every subject with *T* time points, the disagreement between observations *f*(*t*) and *g*(*t*) is split into two aspects: The *absolute systematic error*, that captures the systematic bias between observations and is measured at full-expiration as:

$$\begin{array}{c} ASE(f,g)=|f(T)-g(T)| \end{array}$$ (6)

and the *mean absolute residual error*, that captures the variations once the systematic bias is removed:

$$\begin{array}{c} MARE\left(f,g\right)=\frac{1}{T}\sum_{t=1}^{T}|(f(t)-g(t))-(f(T)-g(T))| \end{array}$$ (7)

**Fig 4:**
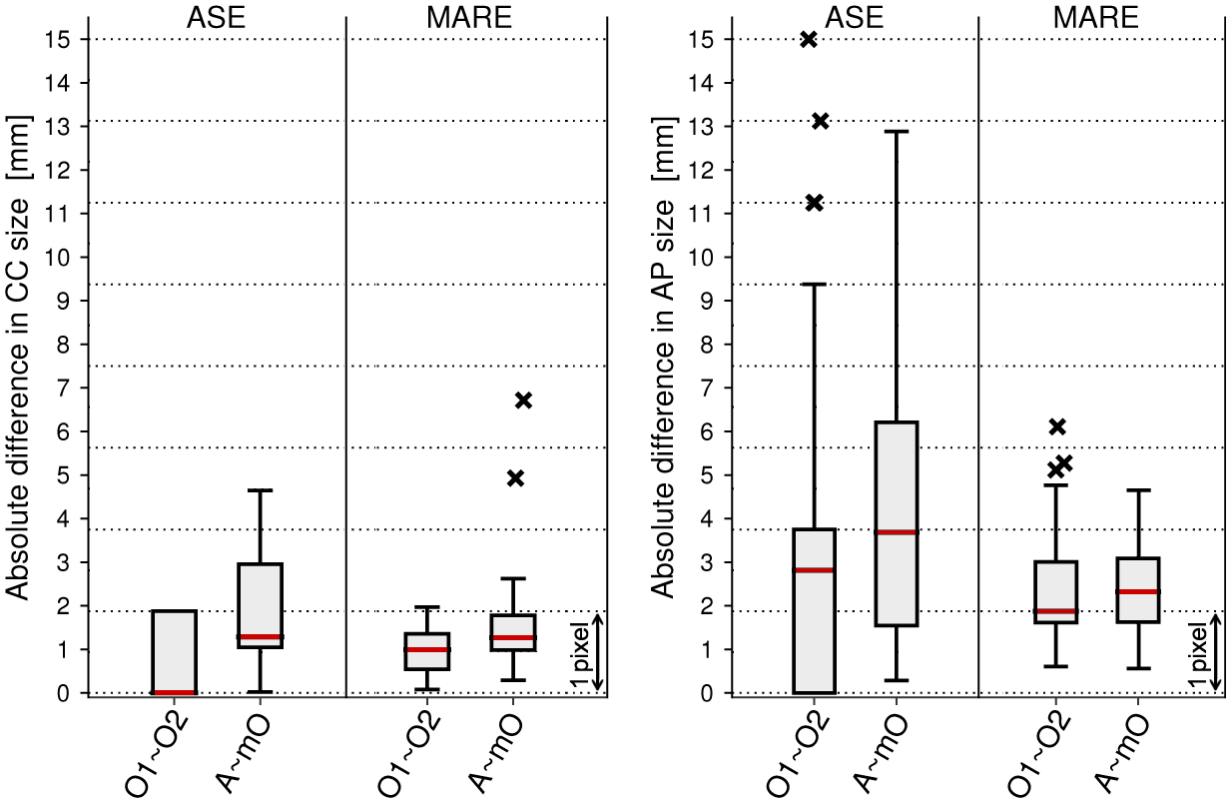
Deviation between manual and automatic lung size computation. The boxplots summarize the ASE and MARE error for CC (left plot) and AP (right plot) lung size measurements. Measurements are compared between observer 1 (O1), observer 2 (O2), mean of observer 1 and 2 (mO), and the automatic method (A). Median values are displayed as horizontal lines in the box, the box limits express the 25th and 75th percentile and outliers are plotted as crosses.

Fig 4 shows the inter-observer difference and the difference between the computer aided method and the manual measurements. When determining the CC size at full-expiration, the two observers differ only marginally (mean 0.8 mm, median 0 mm) indicating that the diaphragm edge is easily identifiable by a human observer. The highest point of the diaphragm is determined slightly differently for the automatic method compared to the manual annotation, which results in a median ASE of 1.3 mm (less than a voxel). In both comparisons the median MARE lies below one voxel (inter-observer: 1 mm, observers vs. automatic method: 1.3 mm). A paired, two-sided Wilcoxon signed rank test with significance level of 5% showed that there were statistically significant differences between the inter-observer variability and the manual vs. automatic error.

The exact and reliable identification of the anterior lung edge is much more difficult due to strong ghosting artifacts occurring at the chest region, resulting in a median inter-observer ASE of 2.8 mm. Median ASE between the automatic method and the observers was 3.7 mm. Median inter-observer MARE was computed as 1.9 mm and median MARE of mean observer vs. automatic method as 2.3 mm. For the AP size measurement, no significant difference was observed between the inter-observer error and the error between the automatic method and the mean observer (*p*_*ASE*_ = 0.26, *p*_*MARE*_ = 0.41).

### Motion features in health and disease

The extracted features were used to find differences between diseased and controls. Table 1 shows the detailed results for the proposed image-based features and the spirometry features. In patients where the diaphragm is severely affected by Pompe disease, almost no change in CC size (**B4**) can be observed during exhalation, indicating an impaired diaphragm. In 6 out of 10 patients the CC size at full-inspiration is less than 10% increased versus the full-expiration state. In contrast, 5 out of 6 controls show an increase in CC size of at least 30%. The increase of AP size (**B5**) is however not significantly different between patients and controls and ranges from 10% to 36%. This indicates that Pompe patients conserve the capabilities to partially inflate the lung using the chest muscles despite having an impaired diaphragm. The CC-AP-ratio (**B6**) shows that 5 out of 6 controls have a larger increase in CC than in AP size, while only 4 out of 10 patients present the same behavior.

**Table 1:** Comparison of spirometry- and image-based features.

ID	Feature	Patients	Controls	Rank sum test
**A2**	costal volume displacement [L]*	1.03 (0.35)	1.30 (0.61)	0 (p = 0.37)
**B1**	residual volume [L]	2.18 (0.64)	2.65 (0.69)	0 (p = 0.15)
**B5**	AP size ratio (insp./exp.)	1.17 (0.05)	1.23 (0.09)	0 (p = 0.15)
**S6**	MEP sitting [%pred]	83.49 (20.06)	104.93 (9.12)	1 (p = 0.04)
**B6**	CC-AP size ratio (**B4**/**B5**)	0.95 (0.11)	1.10 (0.07)	1 (p = 0.0312)
**S5**	MIP sitting [%pred]	74.75 (16.29)	99.49 (19.02)	1 (p = 0.0225)
**S4**	PEF supine [%pred]	61.39 (18.21)	103.15 (29.29)	1 (p = 0.0127)
**A3**	diaphragm contribution to displ. vol. [%]	12.16 (16.68)	41.57 (15.39)	1 (p = 0.0110)
**B4**	CC size ratio (insp./exp.)	1.20 (0.16)	1.35 (0.10)	1 (p = 0.0010)
**A1**	diaphr. volume displacement [L]*	0.15 (0.22)	0.84 (0.22)	1 (p = 0.0010)
**B2**	vital capacity (insp.-exp.) [L]*	1.62 (0.55)	3.29 (0.83)	1 (p = 0.0005)
**B3**	volume ratio (insp./exp.)	1.68 (0.23)	2.42 (0.42)	1 (p = 0.0005)
**A4**	mean CC diaphragm excursion [mm]*	14.32 (16.08)	60.48 (14.42)	1 (p = 0.0005)
**A6**	diaphr. orientation at full-insp. [°]	4.19 (5.25)	21.83 (4.71)	1 (p = 0.0005)
**S1**	FVC supine [%pred]	45.70 (13.03)	100.17 (7.88)	1 (p = 0.0002)
**S1b**	FVC sitting [%pred]	64.00 (14.09)	101.83 (6.21)	1 (p = 0.0002)
**S3**	FEV1 supine [%pred]	40.65 (9.15)	91.63 (14.84)	1 (p = 0.0002)
**A5**	A-P diff. of CC diaphr. excursion [mm]*	-1.68 (1.96)	8.93 (4.12)	1 (p = 0.0002)
**A7**	maximum diaphr. orientation change [°]	3.99 (5.11)	-13.96 (5.57)	1 (p = 0.0002)
**S2**	FVC drop [%pred]	28.95 (11.59)	2.04 (4.02)	1 (p = 0.0002)

Evaluation of basic image features (**B1**—**B6**), advanced image features (**A1**—**A7**) and PFT features (**S1**—**S6**). Mean values and standard deviations are displayed for patients and controls separately. The two-sided Wilcoxon rank sum test (with 5% significance level) indicates significant differences between patient and control group. The table is sorted in descending order by p-value. Normalization with respect to residual volume was performed for features labelled with a star (*).

**Fig 5:**
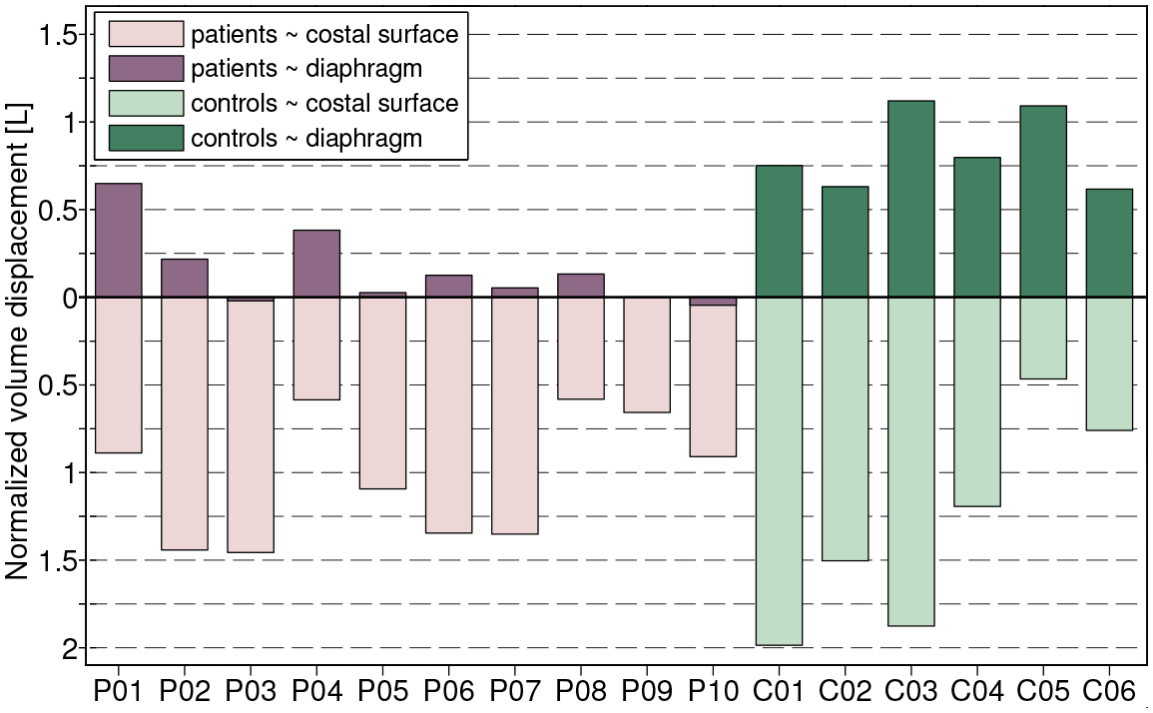
Chest wall and diaphragm contribution to overall lung volume change. The bar plot shows the amount of volume displaced by the costal surface (upper bars) and diaphragm surface (lower bars) for all subjects. Patients (*P01*—*P10*) and controls (*C01*—*C06*) are sorted within their group in descending order with respect to supine FVC (% of predicted).

**Fig 6:**
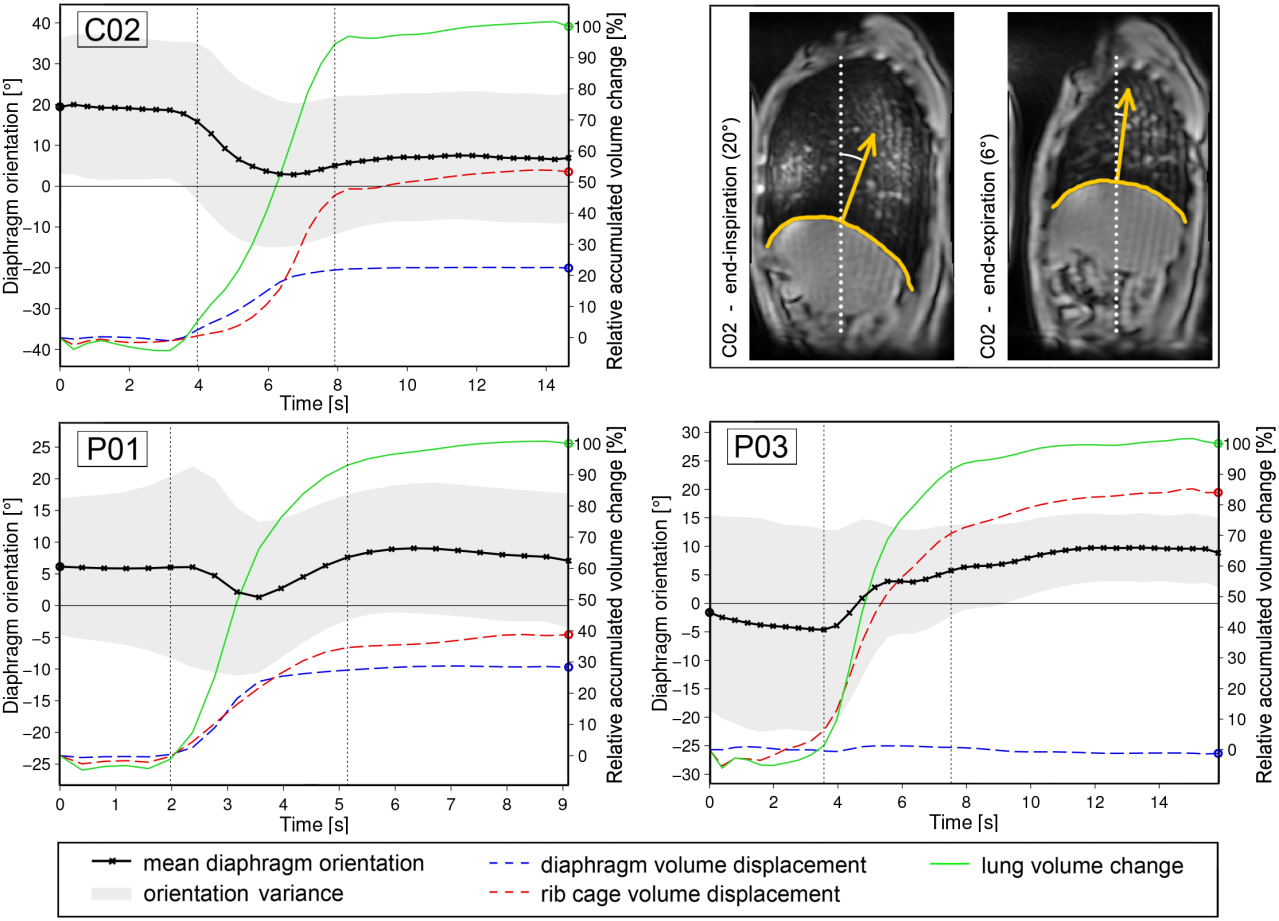
Diaphragm orientation and volume displacement during exhalation. The orientation of the right diaphragm dome (black line: mean value, gray area: variance) is plotted for the exhalation maneuver of two patients (*P01*, *P03*) and a healthy control (*C02*). Start and end of motion are indicated by vertical, dotted lines. Frame-to-frame volume displacement (dashed red line: costal surface, dashed blue line: diaphragm surface) and total lung volume change (solid green line) are accumulated over the sequence and normalized with respect to total lung capacity.

The volume that is displaced by the diaphragmatic surface and the costal surface is presented both integrated over the whole maneuver (Fig 5), and as a time-dependent cumulative increase (Fig 6). In all but one subject (*C05*), the total volume displacement was higher for the costal surface (**A2**) than the (smaller) diaphragmatic surface (**A1**). Fig 5 shows that in 8 out of 10 patients less than 217 ml were displaced by the diaphragm, whereas all controls displaced more than 617 ml. In contrast, the volume displaced by the costal surface is not significantly different between patients and controls, ranging from 581 ml to 1457 ml and 467 ml to 1986 ml respectively. The contribution of the diaphragm to volume change (**A3**) is significantly smaller in patients than in controls. In patients, lung volume changes are thus strongly based on chest wall movement. Table 1 presents further statistics for the features **A1**—**A3**.

In Fig 6 the changing orientation of the diaphragm dome is shown for three selected subjects. Both a representative patient (*P03*) and control (*C02*) are chosen as well as a patient with relatively good respiratory performance (*P01*). As seen in the example of subject *C02*, the diaphragm dome is tilted backwards at full-inspiration in healthy subjects. In the group of controls, the average diaphragm orientation (**A6**) was 22° ± 5° (range: 15°—29°). During exhalation the diaphragm rises and the posterior part of the muscle abuts the chest wall. The angle indicating diaphragm dome orientation thus decreases, resulting in a maximum orientation difference (**A7**) of -14° ± 6° (range: -23°—-8°) in the healthy control group. In Pompe patients the mean orientation at full-inspiration was 4° ± 5° (range: -2°—17°). Compared to the healthy controls, the orientation change during exhalation behaved in the opposite manner for 8 out of 10 patients. On average, the maximum orientation change with respect to the start of exhalation was 4° ± 5° (range: -5°—10°). Patients *P04* and *P01* showed characteristics of both groups (Fig 6). The course of diaphragm orientation was similar to healthy controls but diaphragm orientation at full-inspiration was considerably reduced with respect to healthy subjects.

**Fig 7:**
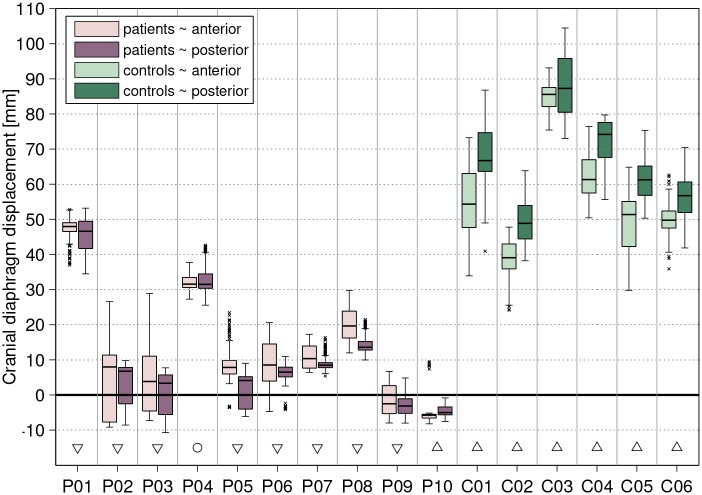
Cranial excursion of the right hemidiaphragm: anterior vs. posterior. The light and dark boxplots for each subject represent the cranial excursion of all points in the anterior and posterior part of the right diaphragm dome, respectively. Significant differences between anterior and posterior excursion are marked with triangles (down-pointing triangle: larger anterior excursion, up-pointing triangle: larger posterior excursion) and circles indicate no significant difference.

At last, the maximum craniocaudal excursion of diaphragm points was investigated. Average cranial displacement (**A4**) in healthy controls was 61 mm (range: 51–81 mm) and 14 mm (range: -4–48 mm) in Pompe patients. The hypothesis that anterior and posterior part of the diaphragm move differently in patients and healthy controls (**A5**), was investigated for the right hemidiaphragm (Fig 7). In accordance with findings from literature, the median displacement was significantly larger in the posterior than in the anterior diaphragm part (difference in the range of 1.7–12.8 mm). The patient group presented the opposite behavior such that displacement is either in the caudal direction (*P10*, *P09*), no significant difference between anterior and posterior diaphragm parts can be observed (*P04*), or the anterior part moves significantly more cranially than the posterior part (*P01*—*P03*, *P05*—*P08*).

**Fig 8:**
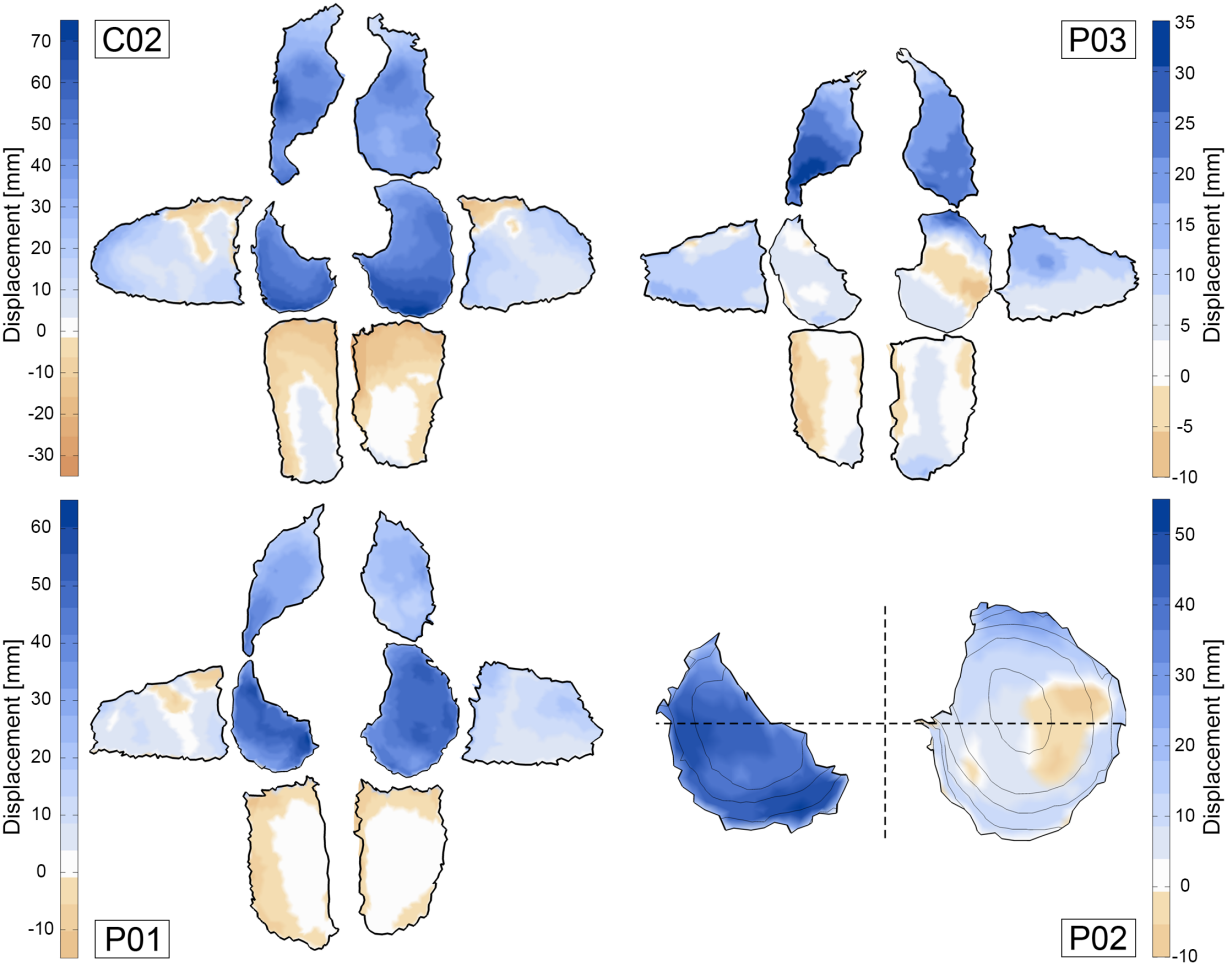
Maximum excursion of points on costal and diaphragm surface. The outer six segments of subjects *C02*, *P01* and *P03* represent the left, right, left/right-anterior and left/right-posterior parts of the costal surface. The maximum amplitude of surface excursion is measured in right, left, posterior and anterior direction, respectively. The left and right diaphragm surface segments of subjects *C02*, *P03*, *P01* and *P02* display cranial excursion. Positive motion (towards lung center) is colored in blue. The dashed lines in *P02* indicate the separation line for the anterior-posterior comparison presented in Fig 7.

The 2D displacement maps in Fig 8 enable the visual investigation of maximal excursion in all relevant lung surface regions. Due to the supine position during the scan, the subjects back is resting on the scanner table which leads to a minimal movement of the posterior costal surface. The three displayed maps represent common motion patterns observed in the study population. Patients with strongly reduced pulmonary function (e.g. *P03*) show less motion in the diaphragm parts than in the anterior costal surface. Even paradoxical motion can occur, which means that diaphragm points move caudally during exhalation (*P03* in Fig 8). In healthy controls, the diaphragmatic surface displaces the most. Especially in the right hemidiaphragm a gradient can be observed with maximum displacement in the posterior and lateral parts, and less displacement in the anterior part. In patient *P01*, overall displacement of the diaphragmatic surface is comparable with the healthy controls. However, the posterior parts in both left and right hemidiaphragm show less movement than the anterior parts.

### MRI vs. PFT measurements

The relation between pre-scan spirometry features (**S1**—**S6**), basic image features (**B1**—**B6**) and advanced image features (**A1**—**A7**) was investigated by computing the Pearson correlation coefficient between all combinations of these features over the 16 subjects. The full matrix of significant correlation coefficients for every pairing of features is shown in S1 Fig. First, note that the spirometry features FVC and FEV1 highly correlate with each other (r = 0.95), indicating that similar clinical conclusions can be drawn from both measurements. The residual volume (**B1**) does not correlate well with any other features, likely because it is highly dependent on the subject’s gender, height and age. However, the image-derived volume ratio (**B3**: TLC/RV) correlates well with FVC (r = 0.88), indicating that both features measure a similar aspect of pulmonary function.

The image-based features related to chest wall movement (**B5**,**A2**) correlated best with MIP (r = 0.67, r = 0.56). Compared to other spirometry features, only poor correlation was observed between MIP and features describing diaphragm motion (r≤0.65). Very high correlation (r = 0.94, r = 0.95, r = 0.97) was found between the image-derived features CC size ratio (**B4**), volume displaced by the diaphragm (**A1**) and mean CC diaphragm excursion (**A4**). All of these features use different computation methods, yet all express the overall diaphragmatic motion, revealing diaphragm impairment in Pompe patients (see Table 1). Of the image-based features, **A1** and **A4** correlate best with spirometry features. However, Table 1 shows that **A5** and **A7** have better discriminative capabilities. This observation suggests that spirometry and image-based features capture different aspects of diaphragm weakness.

## Discussion

With this study we showed that dynamic 3D MRI of the thorax is suitable to evaluate the functional interaction of respiratory muscles during breathing. Computer aided, quantitative analysis of diaphragm motion during slow exhalation was introduced to assess diaphragm weakness and characterize respiratory muscle involvement in patients with Pompe disease. Of the proposed features, diaphragm orientation and displacement difference between anterior and posterior parts of the diaphragm were the features with highest sensitivity to subtle pathophysiological changes.

**Interpretation of results**. In previous studies, PFT was performed in order to assess pulmonary function and involvement of respiratory muscles in Pompe patients [2, 6, 9]. Our image-based results indicate that at an early disease stage patients can compensate for diaphragm weakness with increased chest wall movement leading to relatively normal pulmonary function. For instance, patients *P01* and *P02* exhibit the highest supine FVC in the patient group (69% and 62% of predicted values respectively), however patient *P02* extensively uses rib cage expansion to maximally increase lung volume during inspiration as seen in Fig 5.

The widely proposed analysis of CC and AP lung expansion [30, 35, 43, 54, 55] enables the assessment of general diaphragm and chest wall motion, though we experience that this method is not sensitive enough to detect early pathophysiological changes. For instance, patient *P01* and control subject *C02* both present a CC lung size increase of 30% and comparable AP expansion of 17% and 21%, respectively. By evaluating the diaphragm orientation (Fig 6) and diaphragm displacement feature (Fig 8), moderate diaphragm weakness could be detected in patient *P01*. Note that all proposed image-based features can be obtained for left and right lung separately, e.g. revealing significant different elevation of right and left hemidiaphragm in patient *P02*.

When interpreting the results of the diaphragm features one has to consider that only a part of the diaphragm, the diaphragm dome, can be evaluated with the presented procedure. In addition, according to Suwatanapongched et al. [56], diaphragm shape and position in sitting posture is also related to demographic and physiologic characteristics like weight, age and thoracic dimensions. Gender dependent differences in lung volumes and diaphragm displacement have also been reported previously [19, 57]. One should therefore take this normal variability into account when assessing diaphragm weakness based on geometry features. Furthermore, Kolar et al. [58] showed that diaphragm movement can be induced voluntarily and also non-related to breathing for spine stabilization. This aspect should be taken into account when selecting a breathing maneuver and instructing subjects during the scan.

**Image acquisition procedures in comparison**. Next to dynamic MRI, ultrasound has also been proposed as radiation-free technique to quantitatively evaluate diaphragm kinematics [19, 20]. Some of the frequently presented advantages are its relatively low costs, portability, accessibility, applicability to a wider range of subjects and high temporal resolution. However, in order to assess kinematics of the respiratory system it is necessary to evaluate chest wall and diaphragm motion simultaneously and especially analyze diaphragm motion at defined locations in a reproducible manner, which can be achieved with MRI.

**Motion estimation and lung segmentation**. Multiple strategies have previously been used to segment the lungs in dynamic and/or low resolution static MRI images. Most approaches, like in our pipeline, require the segmentation of a single frame and then adapt this segmentation to fit other frames based on the deformation fields obtained from deformable image registration. In [34] the registration scheme was highly optimized to reduce computation time for tidal breathing sequences by reducing the amount of registrations between frames of very different respiratory phases. The average Dice overlap between the automatic segmentations and ground truth was 0.96. The registration-based segmentation scheme has also been used to segment the lungs in low resolution perfusion images by transforming automatically generated segmentations of high resolution structural images [36]. Comparison with manual segmentations of the perfusion images resulted in a mean Dice overlap of 0.93 ± 1.23. With our pipeline we achieved a mean Dice overlap of 0.87 between the manual segmentation of the full-inhalation (first) frame $M_{1}^{d}$ and the automatic segmentation of the first frame based on the propagation of the manual segmentation of the full-exhalation (last) frame $M_{T}^{d}$. This lower Dice score most likely results from the large range of motion between maximum respiratory phases. The largest overlap error occurs in the area of the heart, the anterior chest wall and the costophrenic recess due to ghosting mainly appearing in these regions.

**Motion and geometry features in healthy subjects**. Studies investigating lung volume and lung dimension changes in healthy subjects between full expiration and full inspiration observed a volume increase of factor 2.5 [24] and 2.09 [35], compared to our observation of 2.42. The increase of craniocaudal/anteroposterior lung size was 1.24/1.18 [35], 1.43/1.27 [54] and 1.56/1.27 [29] which is also comparable to the values from our study (1.35/1.23). In literature, the contribution of the diaphragm to lung volume change has been estimated as 62.5% [30] and 60% [24] whereas our computations suggest a contribution of only 42% in healthy subjects. This is possibly due to different ways of computing the diaphragm contribution. The mean diaphragm excursion in absolute values varies considerably across studies, mostly due to different subject characteristics (especially gender) and different methods of measuring the displacement. Most studies were not spirometer-controlled which increases the dependence on the compliance of subjects. On average, women show less diaphragm excursion and the left hemidiaphragm moves less than the right. The average measurements obtained in our studies, i.e. 6.08 cm, lie in the range of reported values, 6.18 cm [19], 8.17 cm [31] and 8.27 cm [29], whereas the latter two studies only considered male subjects. Our results further confirm the main observation in healthy subjects that the diaphragm tilts backwards during inspiration due to the increased excursion of posterior parts of the diaphragm compared to anterior parts [23–25, 28, 30]. Similar to our study, Vostatek et al. [32] quantify this rotation by approximating the inclination of the manually traced diaphragm dome contour at maximum inspiration (tidal breathing maneuver). The mean inclination in the healthy groups of our study (21.83° ± 4.7°, based on 6 subjects) support the measurements recently made in [32] (23.8° ± 7.1°, based on 16 subjects).

**Image analysis in Pompe disease**. Imaging methods have previously been used in the context of Pompe disease in order to characterize muscle involvement by grading the atrophy in high resolution structural MRI or CT images. In [14] significant trunk muscle involvement was observed except for intercostal muscles. Yet, patients with poorest respiratory function also featured severe intercostal muscle impairment. Gaeta et al. [16] observed prominent diaphragm involvement in most of the patients whereas differences appeared between left and right hemidiaphragm in half of the studied subjects. Abdominal muscles also showed involvement in many patients, yet intercostal muscles were less affected than other muscles. Based on these results, the reduced amplitude of diaphragm excursion observed in our study could be attributed to both inspiratory and expiratory disabilities resulting from diaphragm and abdominal muscle involvement, respectively. The images in [16] also showed significantly reduced lung heights compared to healthy controls and a significant correlation between lung height and diaphragm atrophy grading can be deduced from the data in [16]. This observation supports our approach where diaphragm geometry and excursion are used as surrogate measurement for muscle involvement.

The presented study extends the research by Wens et al. [43], in which static breath-hold MRI images of the same population where acquired at full inspiration and full expiration. Based on manual segmentations, significant differences in CC and AP lung expansion where observed between patients and controls. In a very recent study by Gaeta et al. [55], lung heights (AP and CC) and lung area measurements where manually obtained from 2D static breath-hold MRI images, showing significant differences in CC lung height, diaphragm movement area and lung area measurements between Pompe patients and controls. Both studies show that diaphragm movement is deteriorated in Pompe patients and that patients could compensate for mild diaphragm weakness by increased ribcage expansion. However, our proposed method measures properties of the diaphragm and lung directly and more accurately due to the 3D mesh representation of the entire lungs. Furthermore, intermediate respiratory phases could not be investigated in [43] and [55] and the movement of different parts of the diaphragm was not tracked and evaluated.

**Limitations**. The implemented pipeline currently requires an initial 3D segmentation of one time point of the dynamic scan. In this pilot study, manual segmentations of the static scan were utilized, though multiple publications show that automatic lung segmentation is feasible in 3D MRI [36, 40] or CT [38, 39, 59]. Similarly to the manual step in our pipeline, automatic lung segmentation could be performed utilizing such methods in the high-resolution static scan. The segmentation can then be transformed to the dynamic sequence domain.

Due to the fact that an exhalation maneuver was analyzed in this study, the time-dependent features reflect the relaxation of the diaphragm and efforts of expiratory muscles (mostly abdominal and intercostal muscles) rather than the contraction of inspiratory muscles (diaphragm and intercostal muscles). The proposed analysis should therefore also be applied to a tidal breathing and/or slow full inspiration maneuver, as differences between motion during inspiration and expiration can occur [31]. However, due to the spirometry-controlled scanning procedure used in this work, the frames showing full inspiration are considered to reliably present the state of maximum diaphragm contraction and calculated features can be used to compare to full exhalation frames.

**Future work**. MRI-based analysis presents a promising method which could be used to investigate the optimal start of treatment and to predict treatment response in late-onset Pompe disease. Further research, particularly with a larger and more diverse study population, is necessary to evaluate the whole spectrum of diaphragm involvement and select features which are most sensitive to subtle pathophysiological changes. This feature selection would also require a longitudinal study in order to evaluate their ability to track the disease state. Furthermore, reference values and patterns need to be established for average lung/diaphragm motion based on a larger population of healthy subjects in order to identify abnormal motion in Pompe patients. Future studies should also address the reproducibility of the MRI-based lung motion analysis regarding the breathing maneuvers and automatic image analysis. Both a tidal breathing maneuver and a slow inhalation maneuvers should be recorded in order to analyze diaphragm contraction directly. The proposed methods are applicable to a large variety of medical cases where the diaphragm function itself, the effect of other conditions on the respiratory muscles or general lung deformation needs to be investigated. Other areas of application for this kind of kinematic analysis are, for instance, other neuromuscular diseases affecting the diaphragm, congenital diaphragm hernia, chronic obstructive pulmonary disease, and adolescent idiopathic scoliosis.

## Conclusion

In this paper we have shown that computer aided analysis of 3D+t MRI sequences allows us to in detail quantify respiratory dynamics in healthy and diseased subjects. By using a registration-based segmentation approach, lung surface extraction, and lung surface partitioning, we were able to obtain image-based features that quantify the contribution of the different muscles groups involved in the breathing maneuver. Experimental results show that several of the proposed features, especially features describing the shape change of the diaphragm (including diaphragm orientation and displacement differences within the diaphragm) enable a clear differentiation between Pompe patients and healthy controls. More importantly, the proposed image-based features have been shown to have the potential to be more sensitive to moderate diaphragm weakness than pulmonary function tests. The latter can only measure the combined contribution of all muscles involved with respiration, while image-based analysis can measure the contribution of the different muscle groups separately.

## Supporting Information

S1 Table MRI acquisition parameters. For all MRI acquisitions a 3D SPGR sequence was used with the parameters as specified in the table.
(DOCX)

S2 Table Spirometry- and image-based features of all study participants. The spreadsheet includes measurements from pulmonary function tests and image-based motion features for all patients and healthy controls.
(XLSX)

S1 Video Dynamic MRI scan of a Pompe patient. The video shows a sagittal slice through the right lung of a Pompe patient during a slow exhalation maneuver.
(GIF)

S2 Video Dynamic MRI scan of a healthy control. The video shows a sagittal slice through the right lung of a healthy control during a slow exhalation maneuver.
(GIF)

S1 Data Lung meshes of a Pompe patient and a healthy control. The archive contains a STL file for every time point of the slow exhalation maneuver for a healthy control and a Pompe patient. The files are in VisCAM/SolidView format and contain the lung meshes including color information which indicates the different lung surface partitions.
(ZIP)

S1 Fig Feature correlation matrix. The correlation matrix shows the Pearson correlation coefficient (*ρ*) for feature pairs with significant correlation (p<0.05). Cells with non-significant correlation are rendered in plain white.
(TIF)

S2 Fig Diaphragm orientation diagrams for all study participant. Further explanations are contained in Fig 6.
(PDF)

S3 Fig Maximum excursion color maps for all study participant. Further explanations are contained in Fig 8.
(PDF)

S1 File Elastix parameter files. The archive contains the parameter files used to estimate the forward and inverse transformations. Both files are based on the parameter files which where introduced by Metz et al. [42] and published on http://elastix.bigr.nl/wiki/index.php/Par0012.
(ZIP)
