# Supplementary material for: Quantification of Diaphragm Mechanics in Pompe Disease Using Dynamic 3D MRI
Source: PLoS One. 2016 Jul 8;11(7):e0158912. doi: 10.1371/journal.pone.0158912 (PMC4938606; doi:10.1371/journal.pone.0158912)
Supplement: S1 Table — For all MRI acquisitions a 3D SPGR sequence was used with the parameters as specified in the table. (DOCX) [file pone.0158912.s001.docx]

|  | Static scan | Dynamic scan |
| --- | --- | --- |
| TR/TE | 1.3/0.5 ms | 1.5/0.9 ms |
| Flip angle | 2° | 2° |
| Plane orientation | sagittal volume acquisition | sagittal volume acquisition |
| FOV | 360 mm^2^ | 480 mm^2^ |
| Matrix | 100 x 128 | 100 x 80 |
| Slice thickness | 3 mm | 12 mm |
| Acquired resolution | 3.6 x 2.8 x 3 mm^3^ | 4.8 x 6.0 x 12.0 mm^3^ |
| Interpolated resolution | 1.4 x 1.4 x 1.5 mm^3^ | 1.9 x 1.9 x 6.0 mm^3^ |
| Number of phases |  | 48 volumes in 21 seconds |
| Acquisition options | - Zero-Interpolation filling (ZIP2@General Electric)  - 3D gradwarp option (General Electric) | |

**S1 Table:** **MRI acquisition parameters.** For all MRI acquisitions a 3D SPGR sequence was used with the following parameters.
