# Supplementary figures and images for: Quantification of Diaphragm Mechanics in Pompe Disease Using Dynamic 3D MRI
Source: PLoS One. 2016 Jul 8;11(7):e0158912. doi: 10.1371/journal.pone.0158912 (PMC4938606; doi:10.1371/journal.pone.0158912)

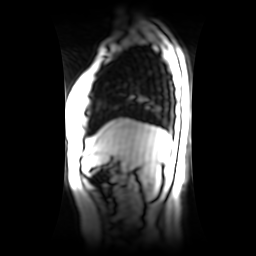

Supplement: S1 Video — The video shows a sagittal slice through the right lung of a Pompe patient during a slow exhalation maneuver. (GIF) [file pone.0158912.s003.gif]

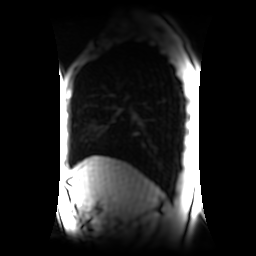

Supplement: S2 Video — The video shows a sagittal slice through the right lung of a healthy control during a slow exhalation maneuver. (GIF) [file pone.0158912.s004.gif]

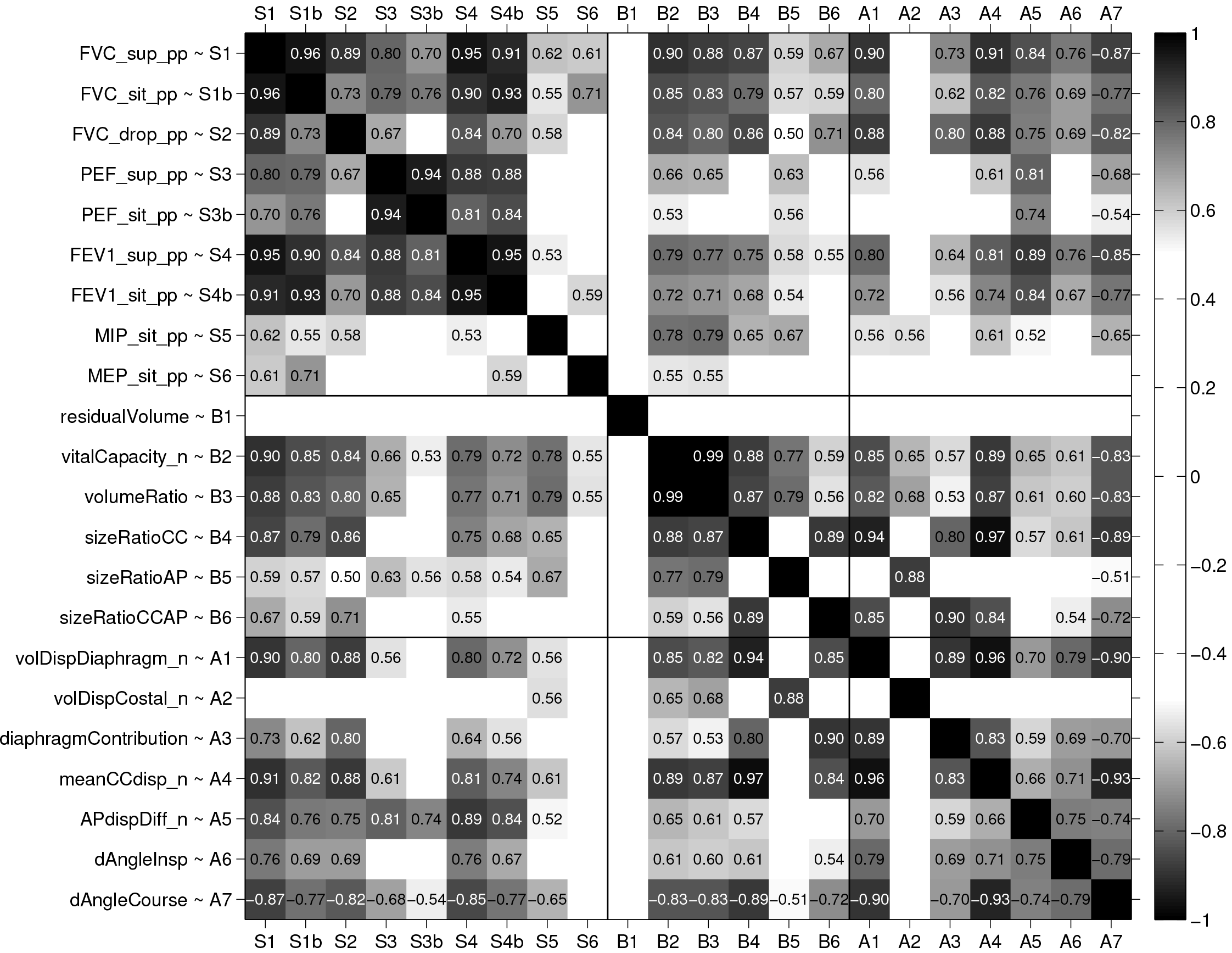

Supplement: S1 Fig — The correlation matrix shows the Pearson correlation coefficient (ρ) for feature pairs with significant correlation (p<0.05). Cells with non-significant correlation are rendered in plain white. (TIF) [file pone.0158912.s006.tif]
