# Supplementary material for: Quantification of Diaphragm Mechanics in Pompe Disease Using Dynamic 3D MRI
Source: PLoS One. 2016 Jul 8;11(7):e0158912. doi: 10.1371/journal.pone.0158912 (PMC4938606; doi:10.1371/journal.pone.0158912)
Supplement: S2 Fig — Further explanations are contained in Fig 6. (PDF) [file pone.0158912.s007.pdf]

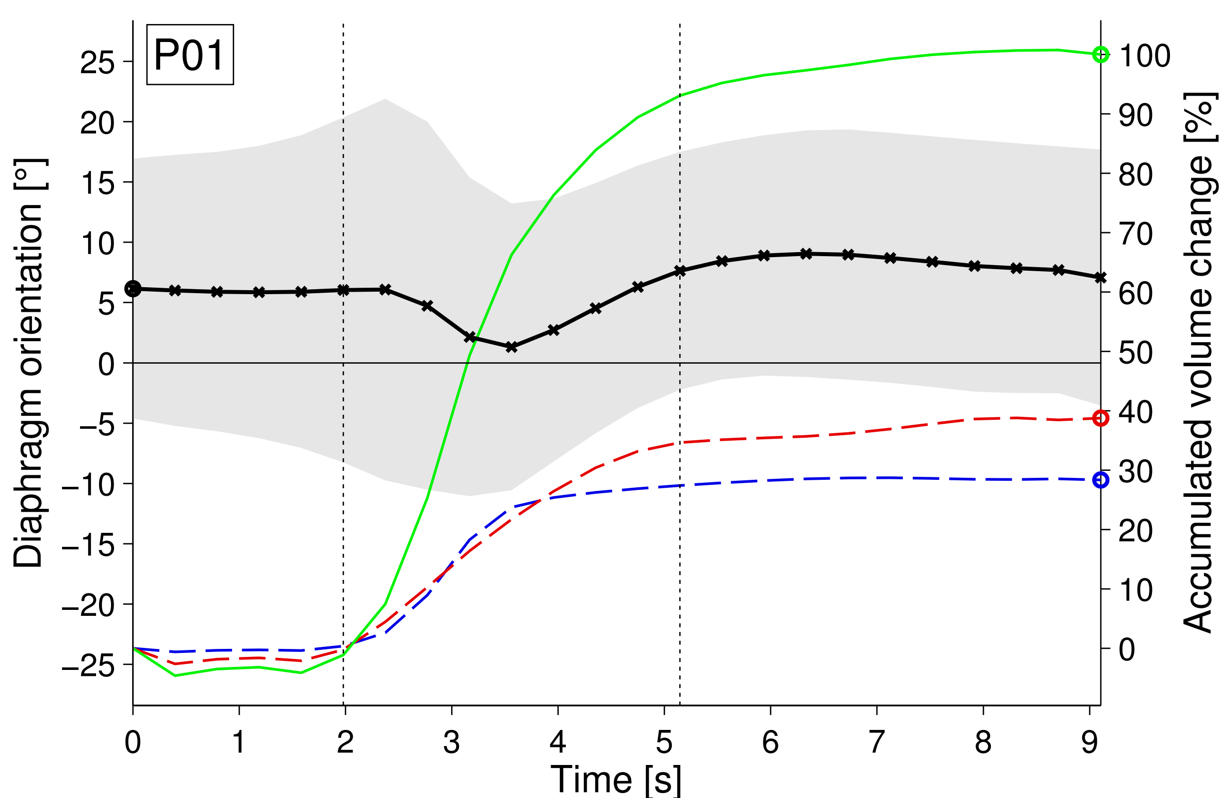

P01
Diaphragm orientation [°]
Accumulated volume change [%]
Time [s]
25
20
15
10
5
0
−5
−10
−15
−20
−25
100
90
80
70
60
50
40
30
20
10
0
0
1
2
3
4
5
6
7
8
9

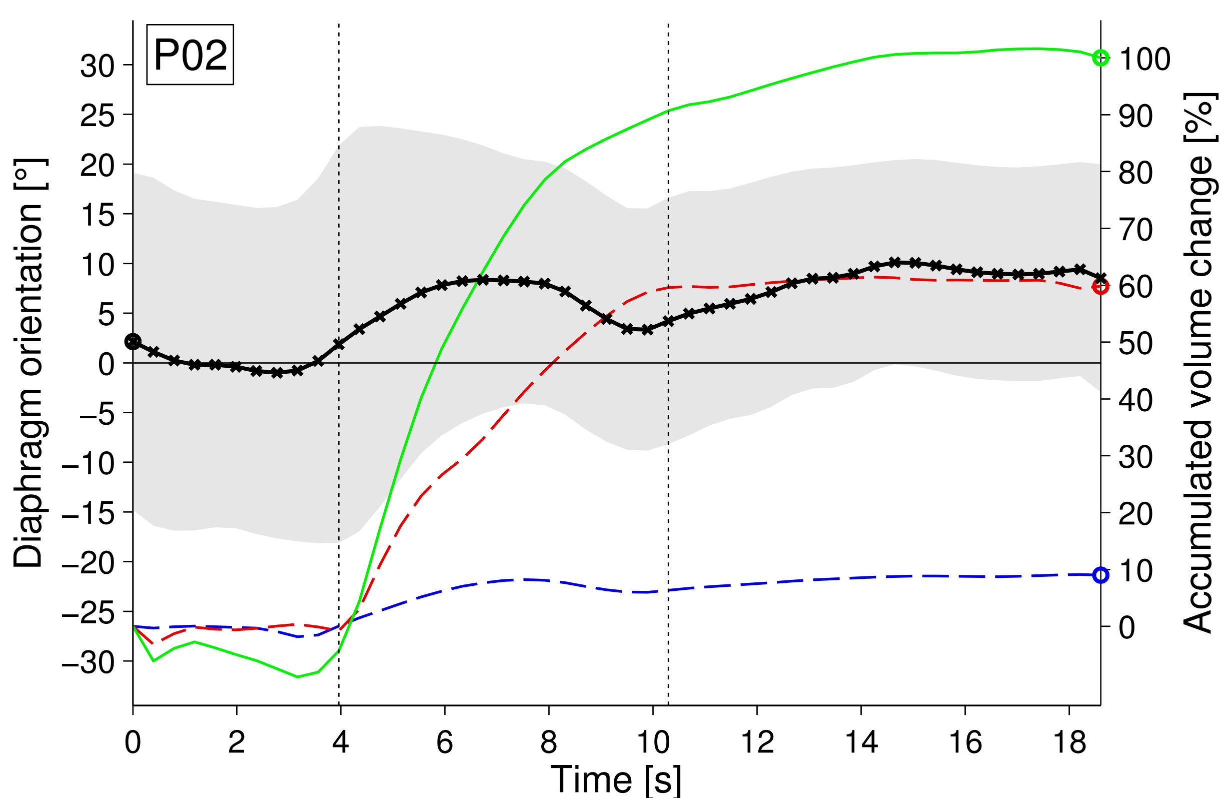
P02
Diaphragm orientation [°]
Accumulated volume change [%]
Time [s]
30
25
20
15
10
5
0
−5
−10
−15
−20
−25
−30
100
90
80
70
60
50
40
30
20
10
0
2
4
6
8
10
12
14
16
18

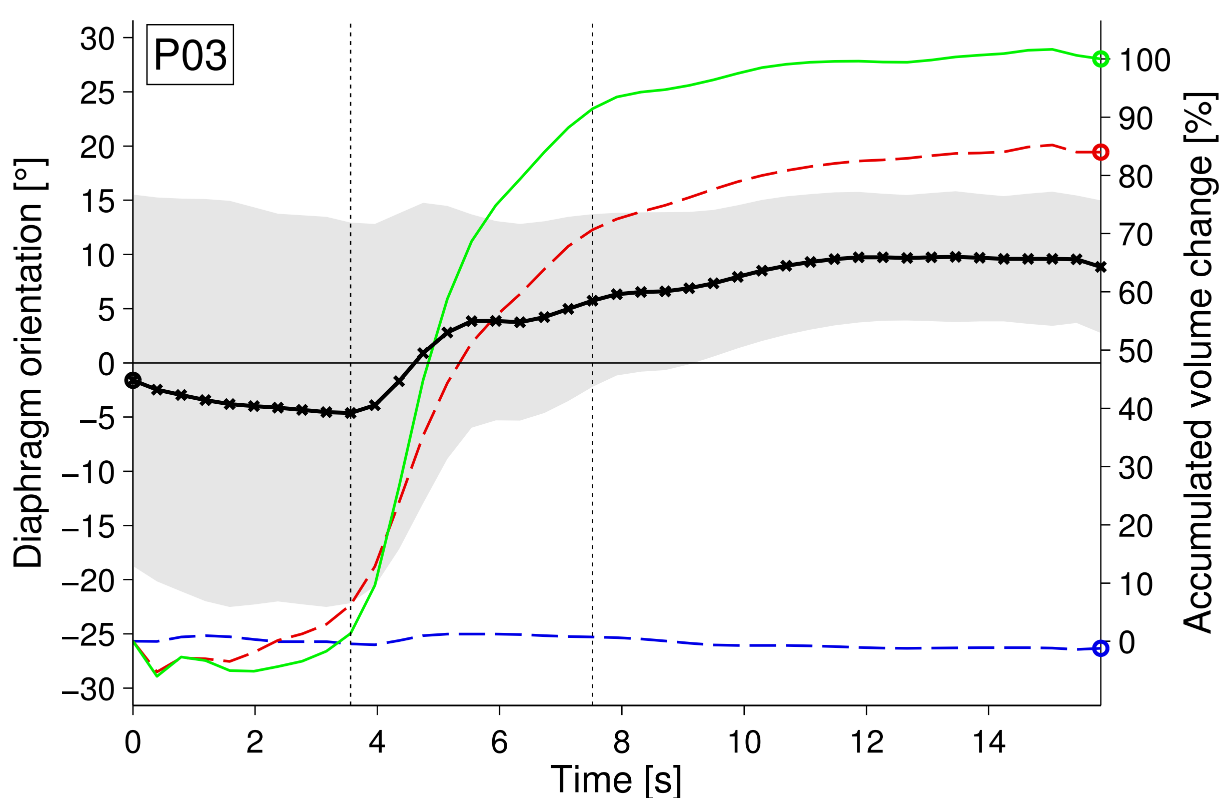

P03
Diaphragm orientation [°]
Accumulated volume change [%]
Time [s]
30
25
20
15
10
5
0
−5
−10
−15
−20
−25
−30
100
90
80
70
60
50
40
30
20
10
0
2
4
6
8
10
12
14

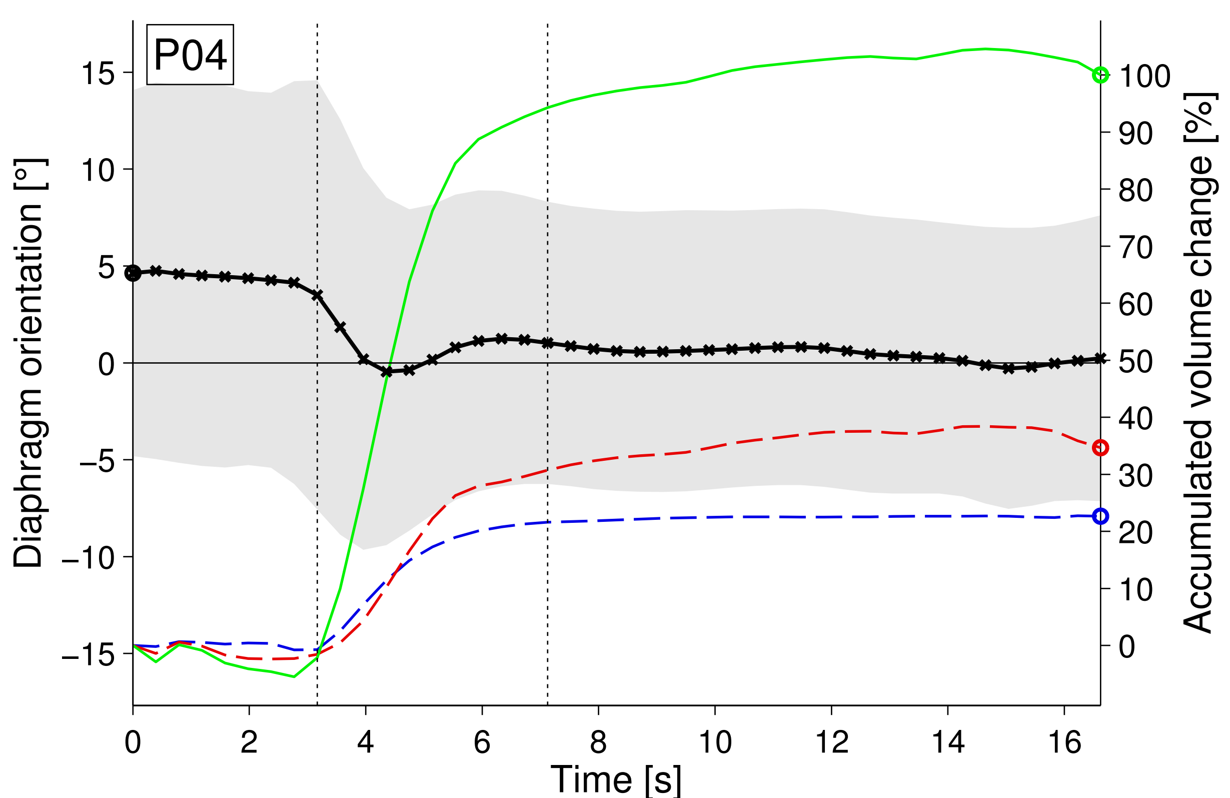
P04
Diaphragm orientation [°]
Accumulated volume change [%]
Time [s]
15
10
5
0
−5
−10
−15
100
90
80
70
60
50
40
30
20
10
0
2
4
6
8
10
12
14
16

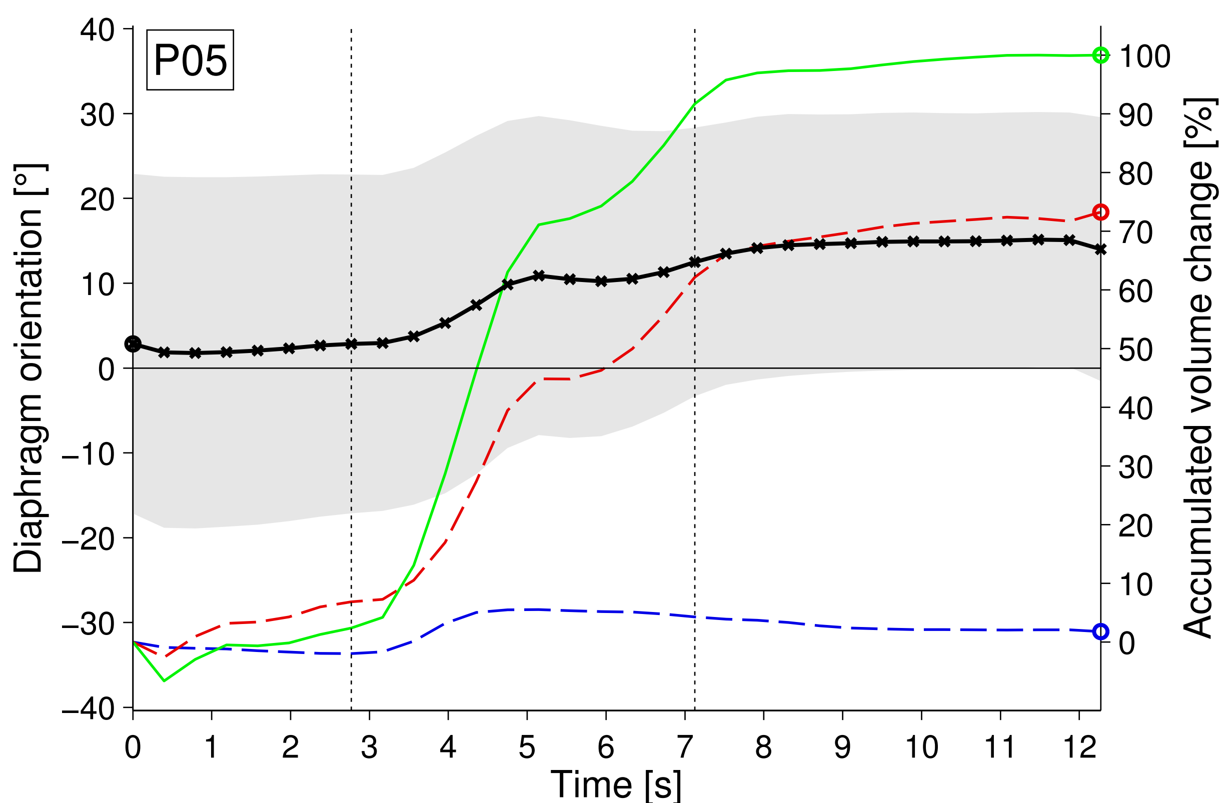
P05
Diaphragm orientation [°]
Accumulated volume change [%]
Time [s]
40
30
20
10
0
−10
−20
−30
−40
100
90
80
70
60
50
40
30
20
10
0
0
1
2
3
4
5
6
7
8
9
10
11
12

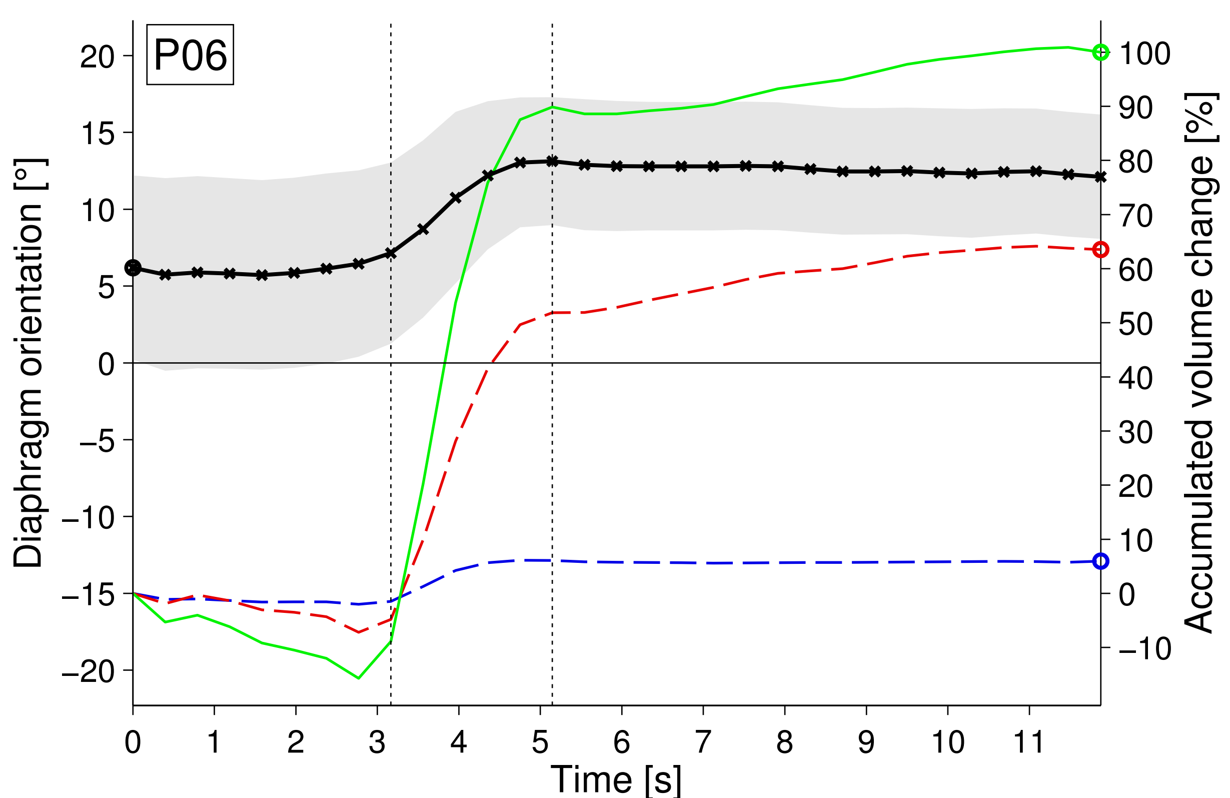
P06
Diaphragm orientation [°]
Accumulated volume change [%]
Time [s]
20
15
10
5
0
−5
−10
−15
−20
100
90
80
70
60
50
40
30
20
10
0
−10
0
1
2
3
4
5
6
7
8
9
10
11

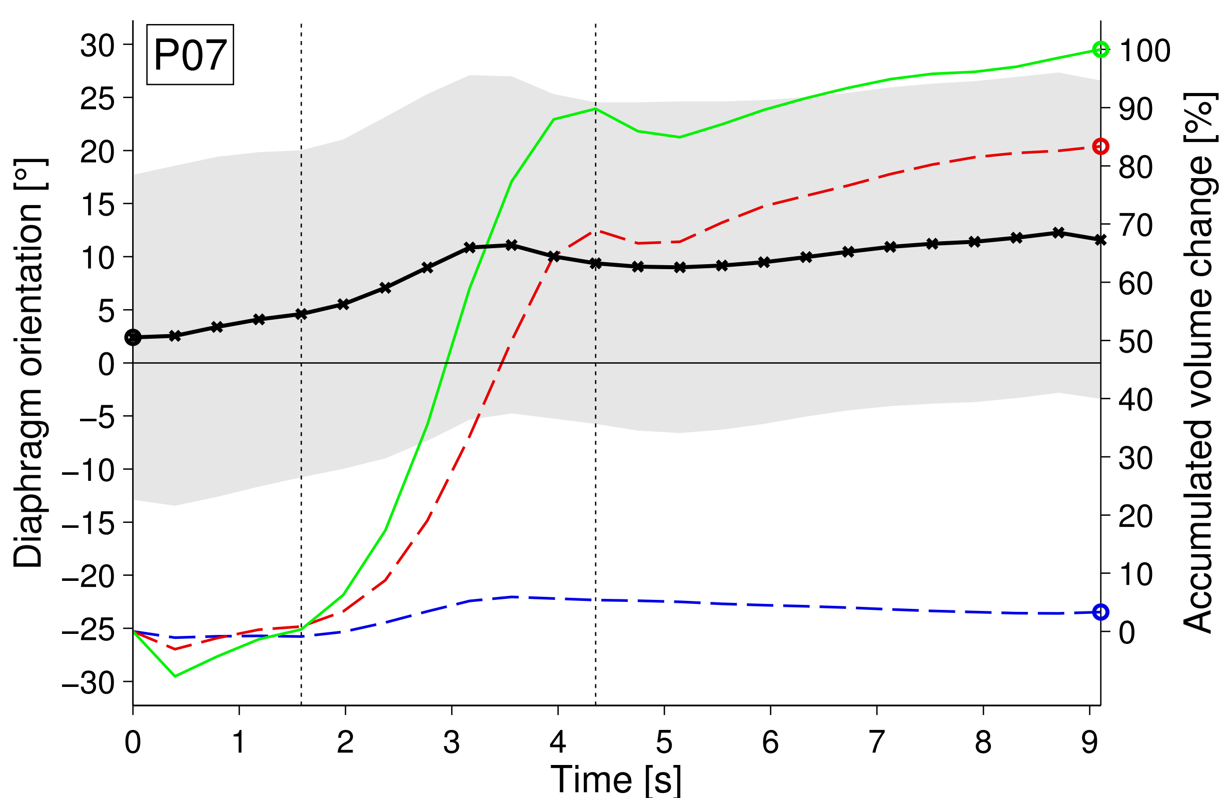

P07
Diaphragm orientation [°]
Accumulated volume change [%]
Time [s]
30
25
20
15
10
5
0
−5
−10
−15
−20
−25
−30
100
90
80
70
60
50
40
30
20
10
0
0
1
2
3
4
5
6
7
8
9

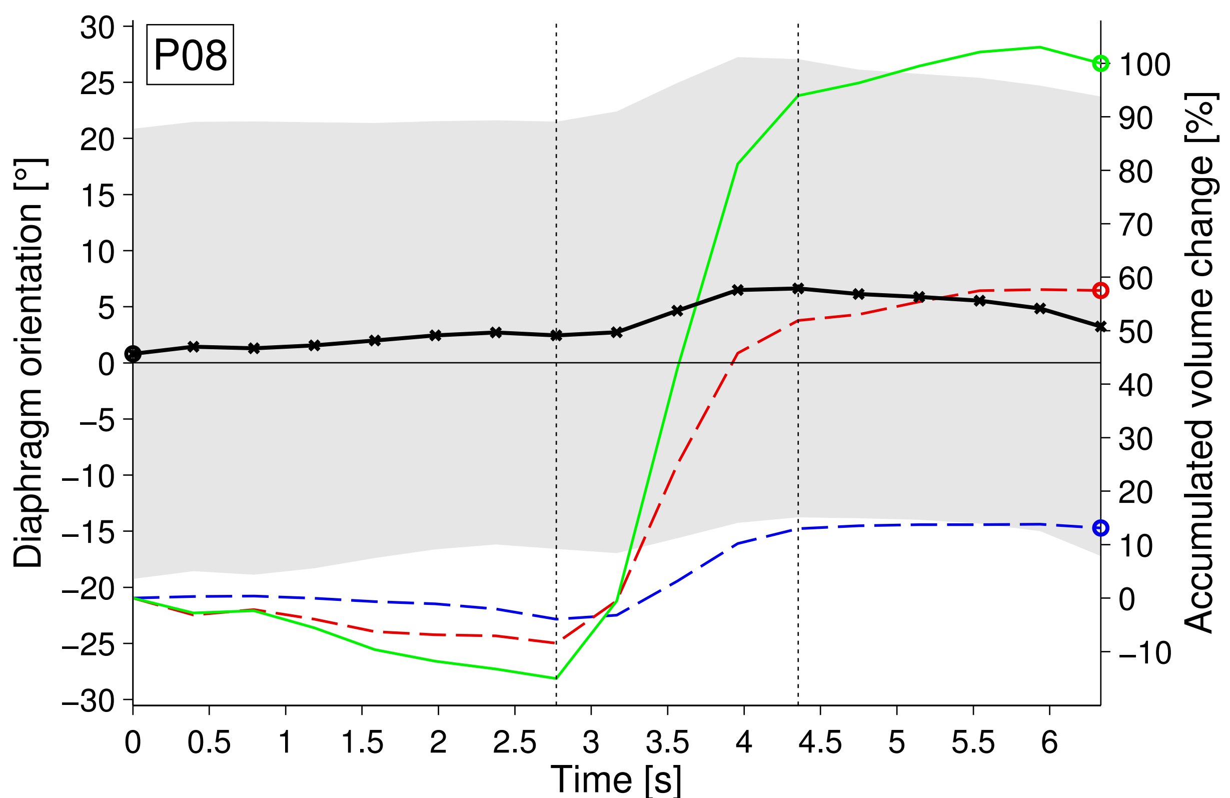

P08
Diaphragm orientation [°]
Accumulated volume change [%]
Time [s]
30
25
20
15
10
5
0
−5
−10
−15
−20
−25
−30
100
90
80
70
60
50
40
30
20
10
0
−10
0
0.5
1
1.5
2
2.5
3
3.5
4
4.5
5
5.5
6

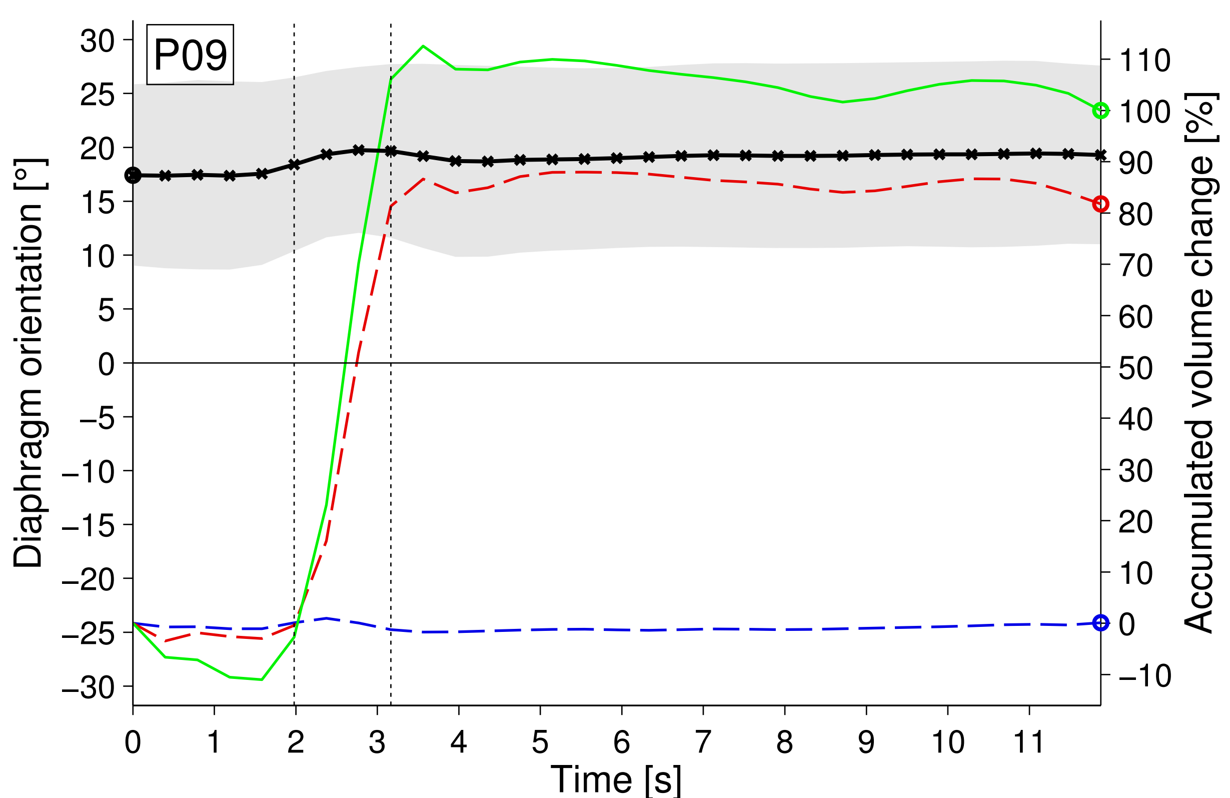

P09
Diaphragm orientation [°]
Accumulated volume change [%]
Time [s]
30
25
20
15
10
5
0
−5
−10
−15
−20
−25
−30
110
100
90
80
70
60
50
40
30
20
10
0
−10
0
1
2
3
4
5
6
7
8
9
10
11

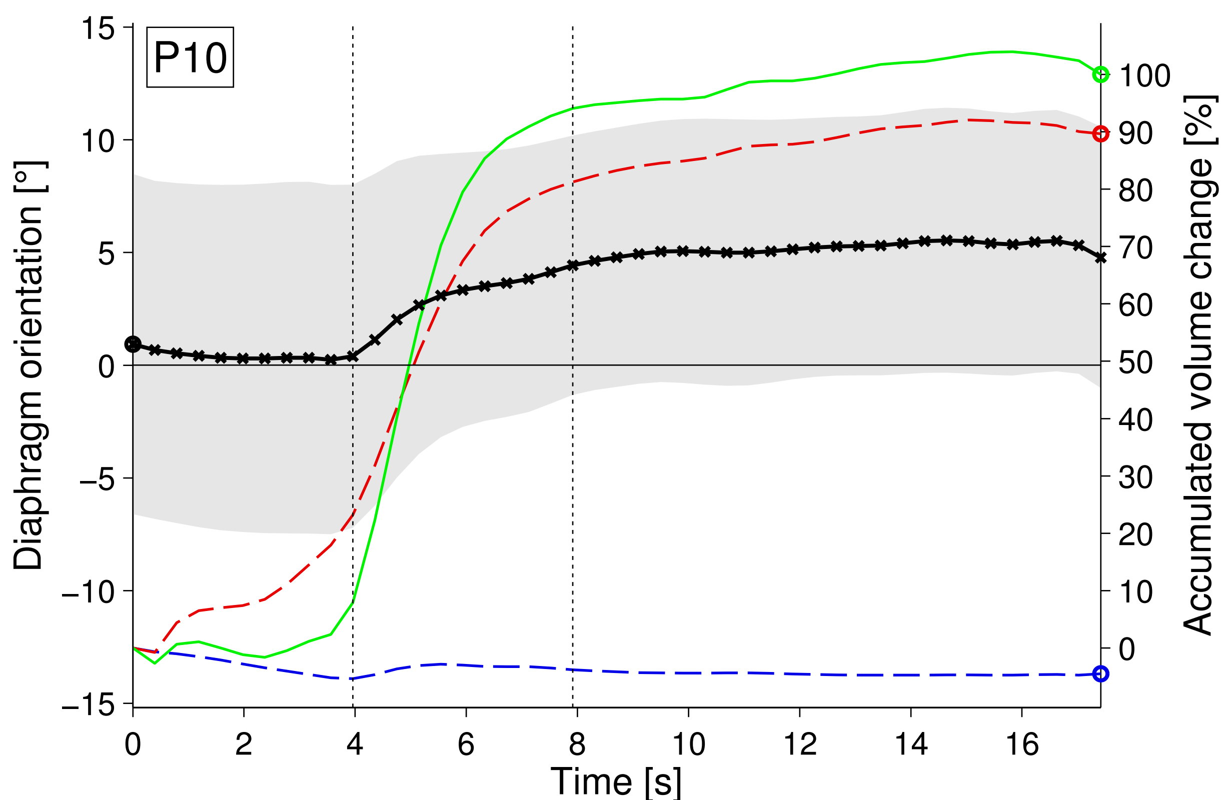

P10
Diaphragm orientation [°]
Accumulated volume change [%]
Time [s]
15
10
5
0
−5
−10
−15
100
90
80
70
60
50
40
30
20
10
0
0
2
4
6
8
10
12
14
16

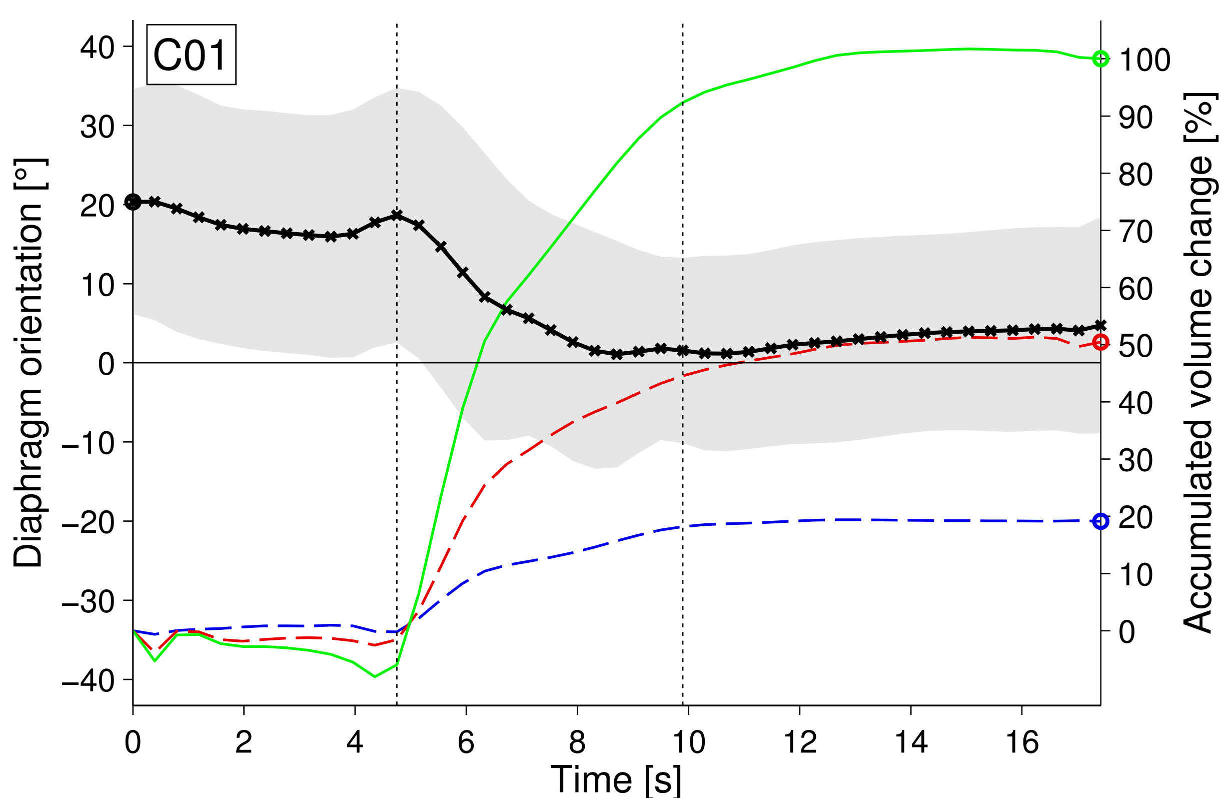
C01
Diaphragm orientation [°]
40
30
20
10
0
−10
−20
−30
−40
Accumulated volume change [%]
100
90
80
70
60
50
40
30
20
10
0
Time [s]
0
2
4
6
8
10
12
14
16

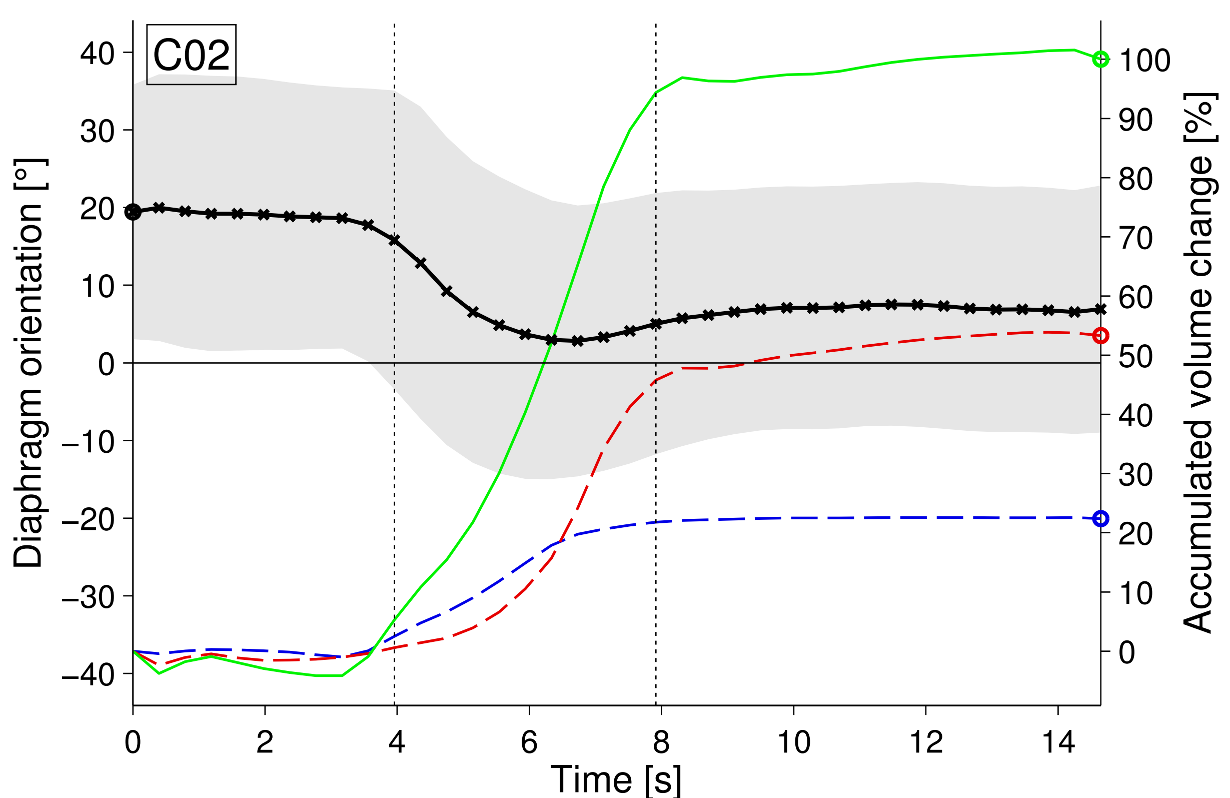

C02
Diaphragm orientation [°]
Accumulated volume change [%]
Time [s]
40
30
20
10
0
−10
−20
−30
−40
100
90
80
70
60
50
40
30
20
10
0
2
4
6
8
10
12
14

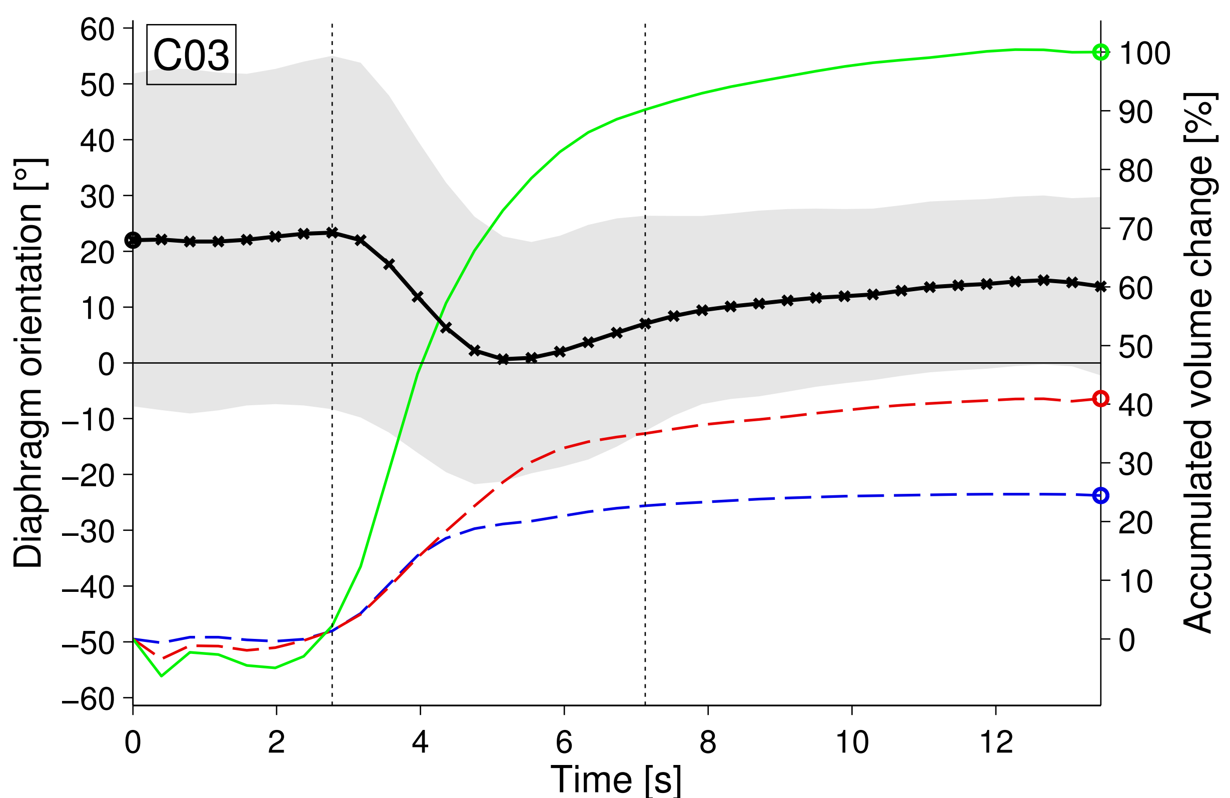

C03
Diaphragm orientation [°]
Accumulated volume change [%]
Time [s]
60
50
40
30
20
10
0
−10
−20
−30
−40
−50
−60
100
90
80
70
60
50
40
30
20
10
0
0
2
4
6
8
10
12

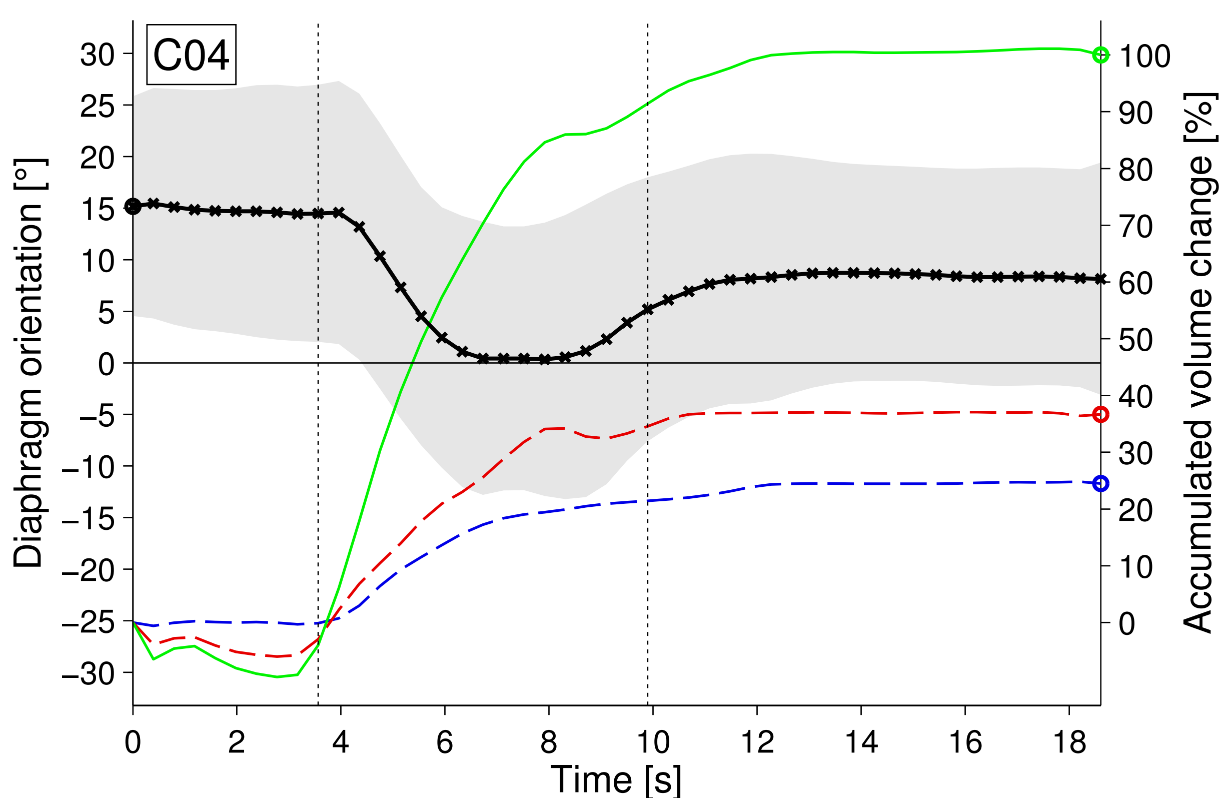

C04
Diaphragm orientation [°]
Accumulated volume change [%]
Time [s]
30
25
20
15
10
5
0
−5
−10
−15
−20
−25
−30
100
90
80
70
60
50
40
30
20
10
0
0
2
4
6
8
10
12
14
16
18

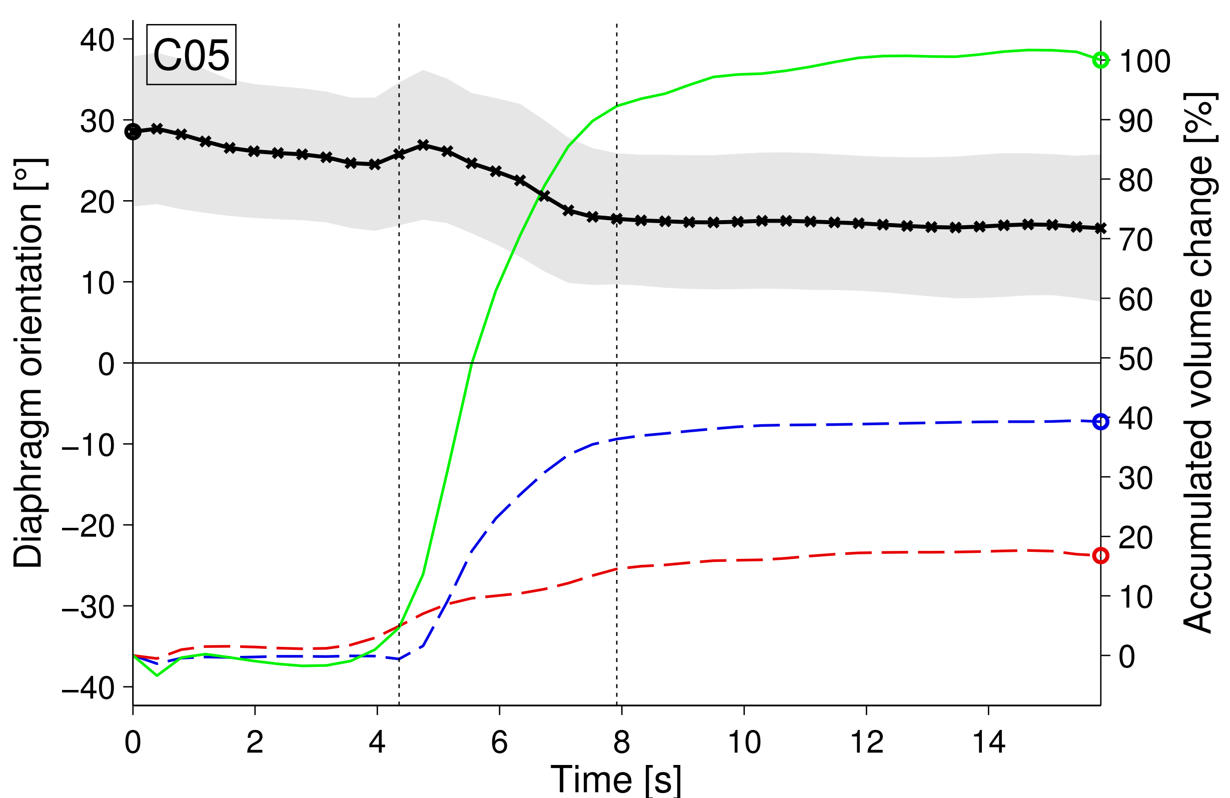
C05
Diaphragm orientation [°]
40
30
20
10
0
−10
−20
−30
−40
Accumulated volume change [%]
100
90
80
70
60
50
40
30
20
10
0
Time [s]
0
2
4
6
8
10
12
14

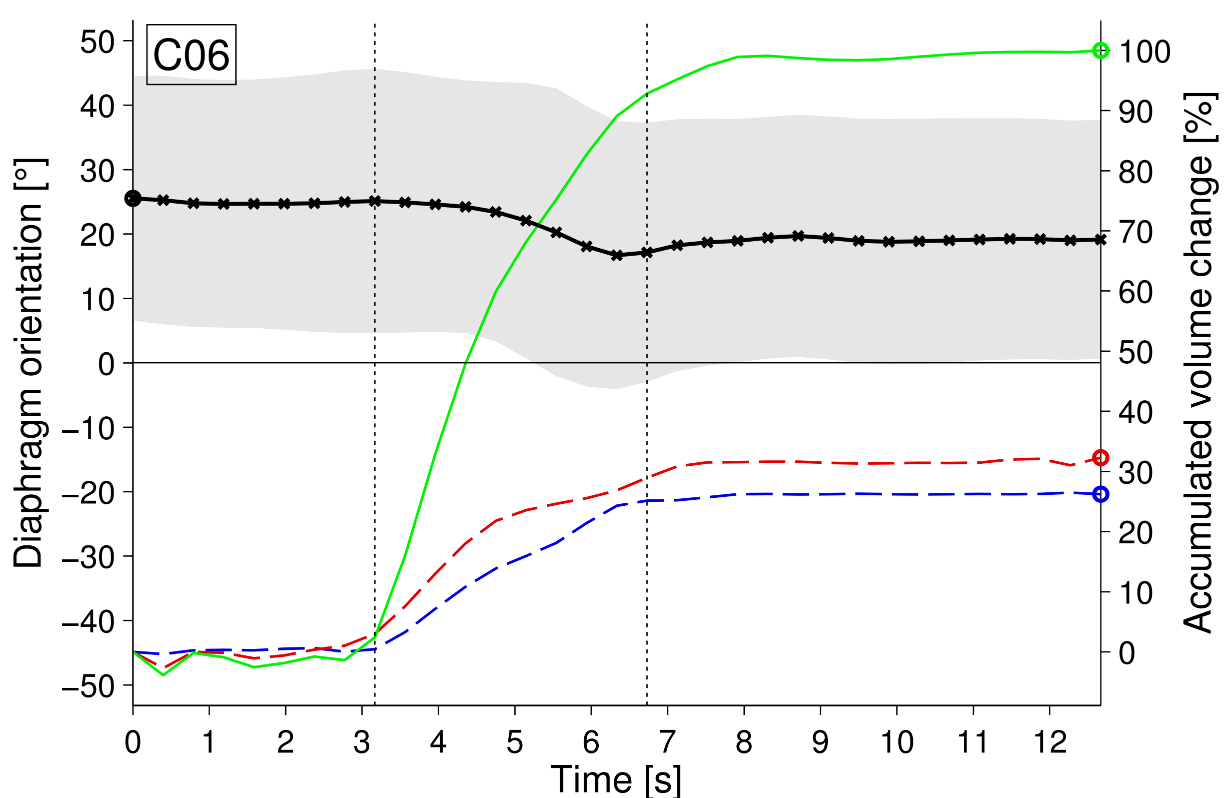
C06
Diaphragm orientation [°]
Accumulated volume change [%]
Time [s]
50
40
30
20
10
0
−10
−20
−30
−40
−50
100
90
80
70
60
50
40
30
20
10
0
0
1
2
3
4
5
6
7
8
9
10
11
12
