# Supplementary material for: Quantification of Diaphragm Mechanics in Pompe Disease Using Dynamic 3D MRI
Source: PLoS One. 2016 Jul 8;11(7):e0158912. doi: 10.1371/journal.pone.0158912 (PMC4938606; doi:10.1371/journal.pone.0158912)
Supplement: S3 Fig — Further explanations are contained in Fig 8. (PDF) [file pone.0158912.s008.pdf]

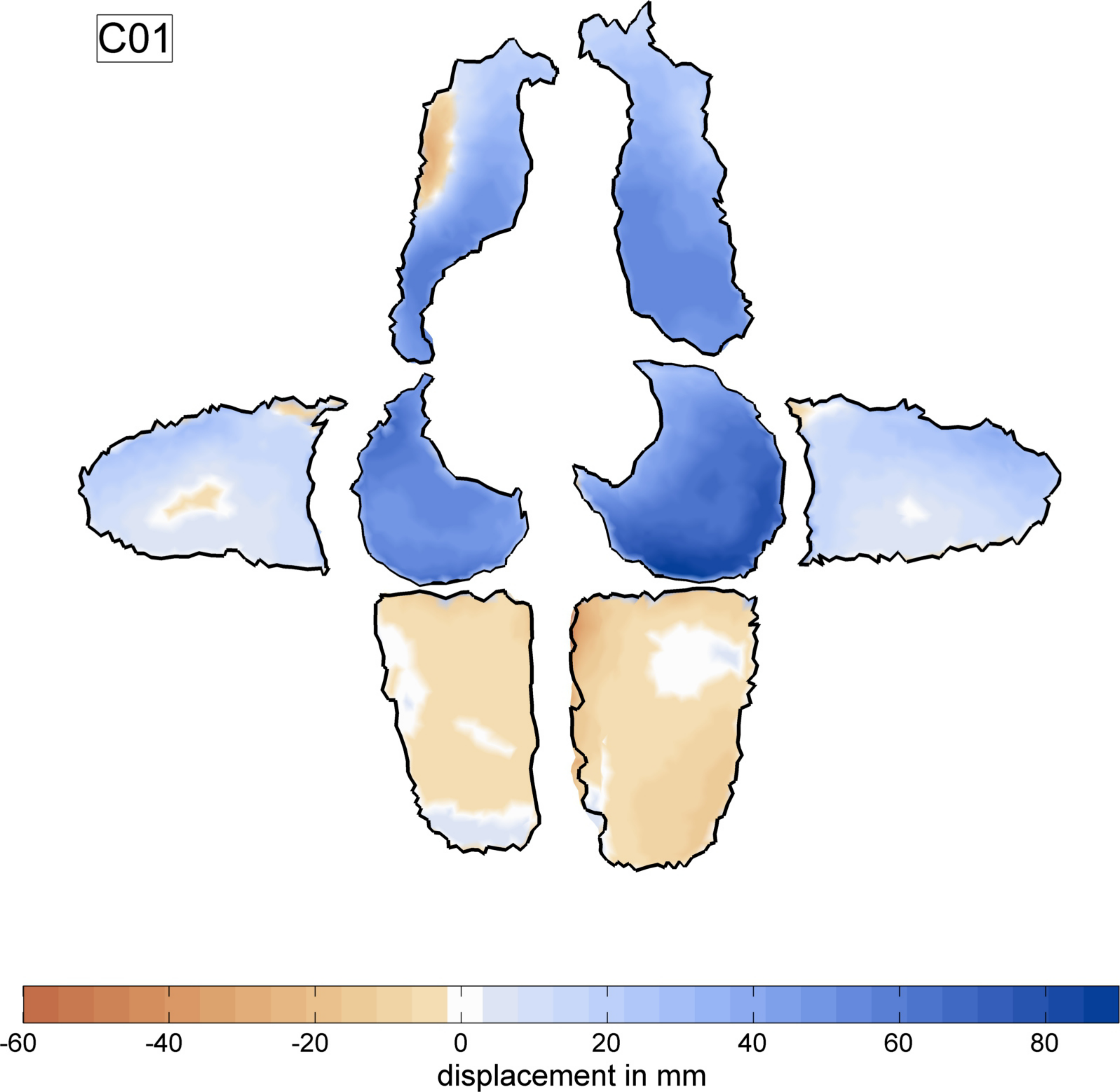
C01
-60
-40
-20
0
20
40
60
80
displacement in mm

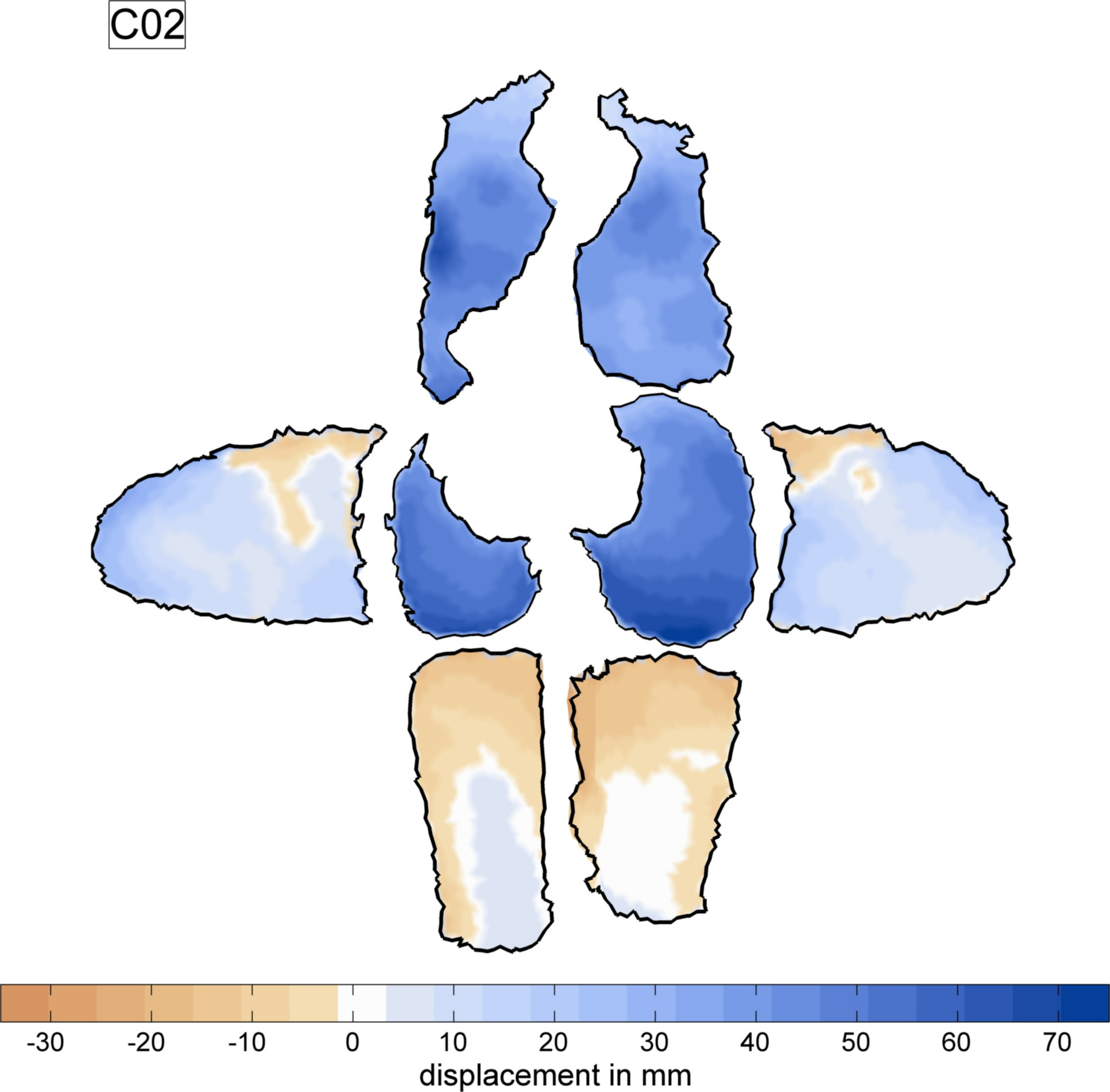
C02
-30
-20
-10
0
10
20
30
40
50
60
70
displacement in mm

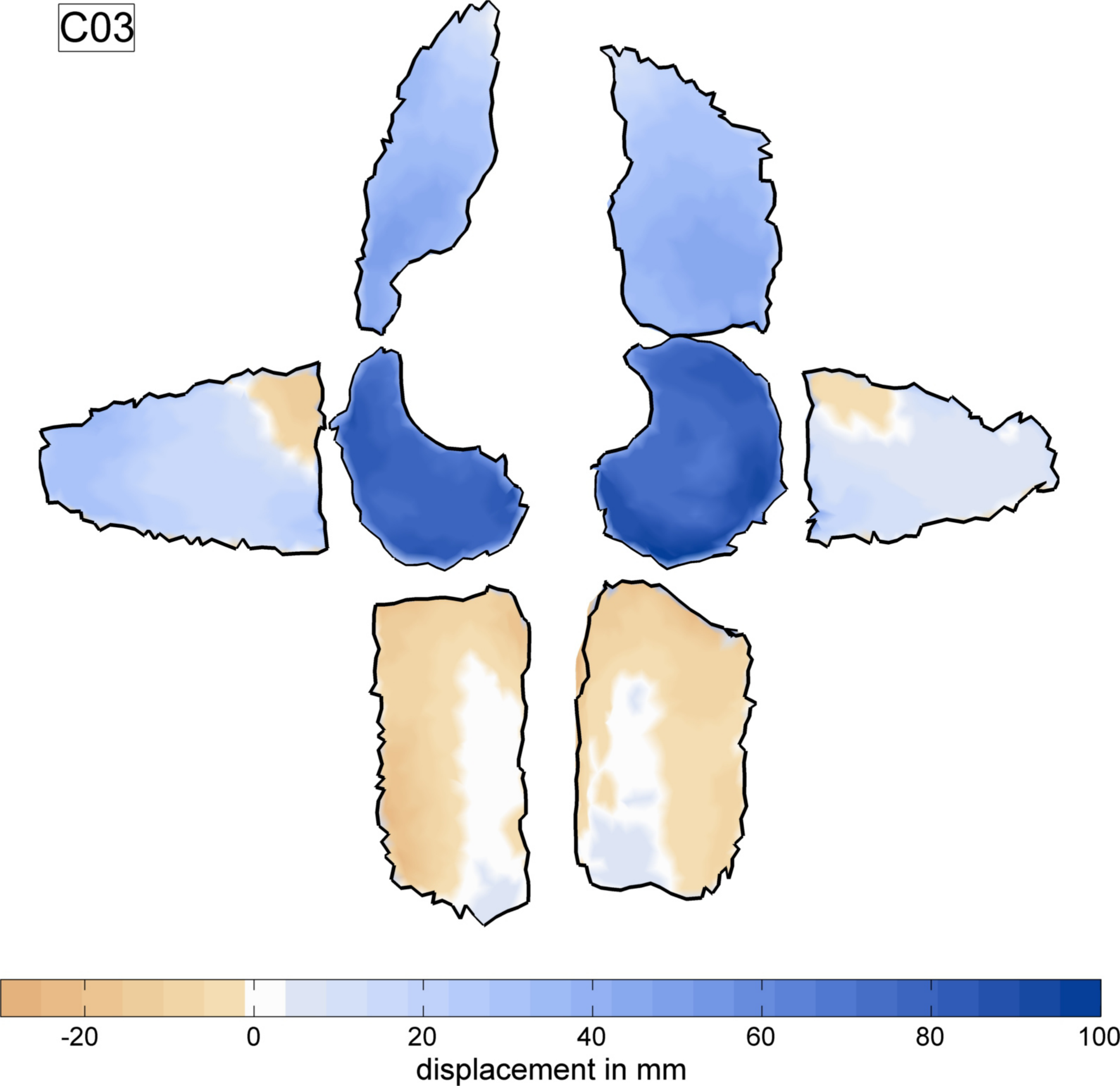
C03
-20
0
20
40
60
80
100
displacement in mm

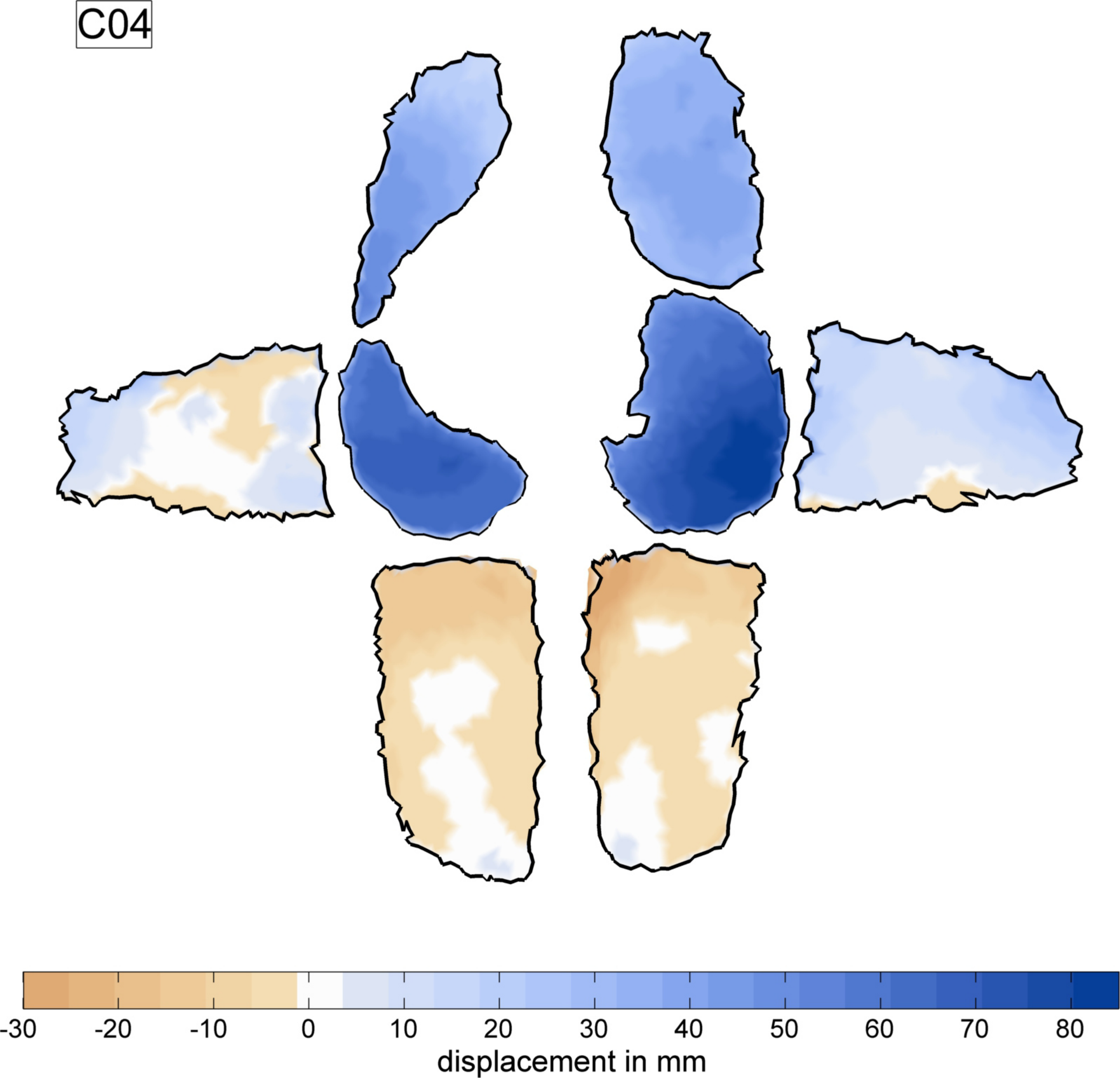
C04
-30
-20
-10
0
10
20
30
40
50
60
70
80
displacement in mm

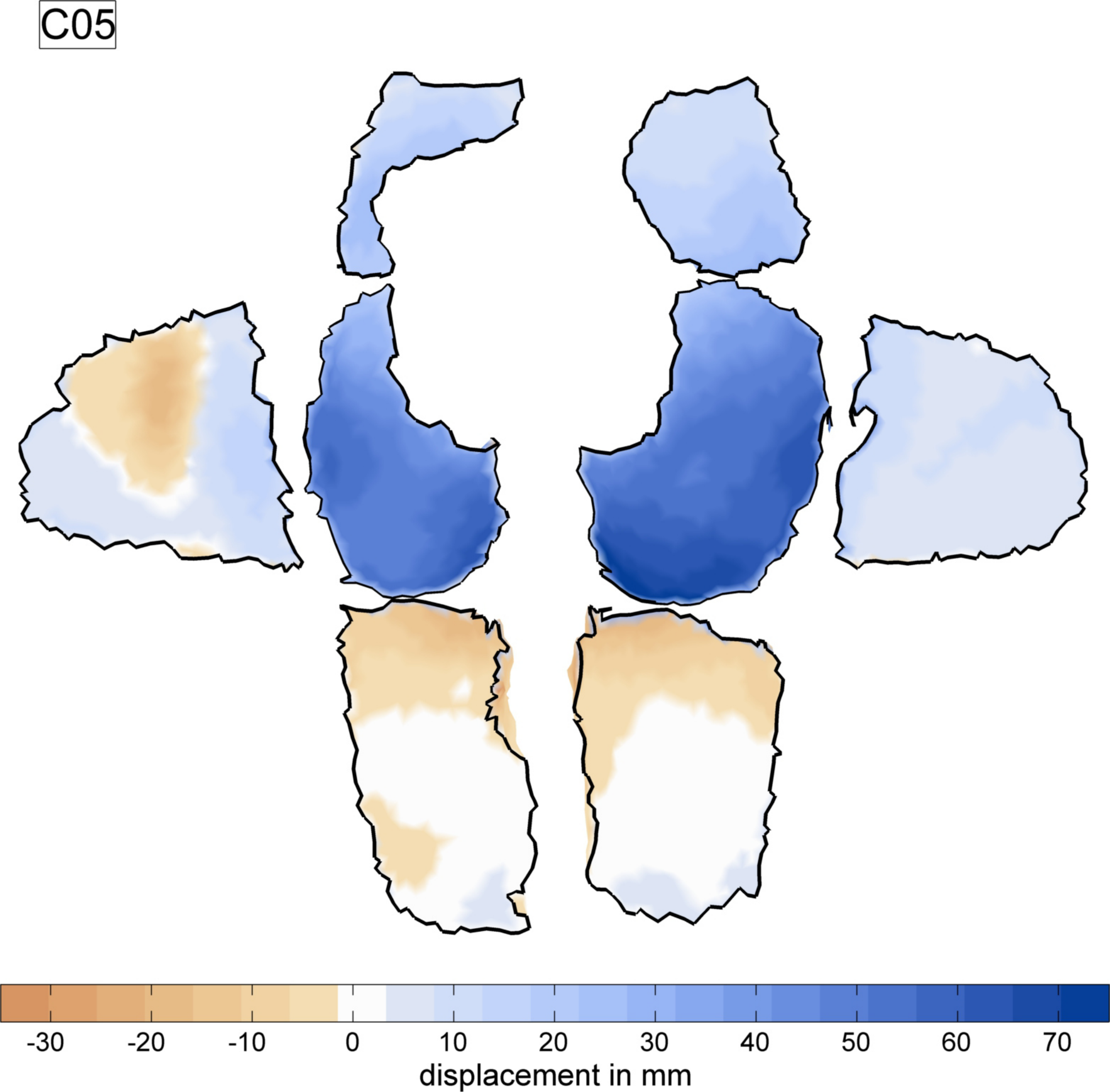
C05
-30
-20
-10
0
10
20
30
40
50
60
70
displacement in mm

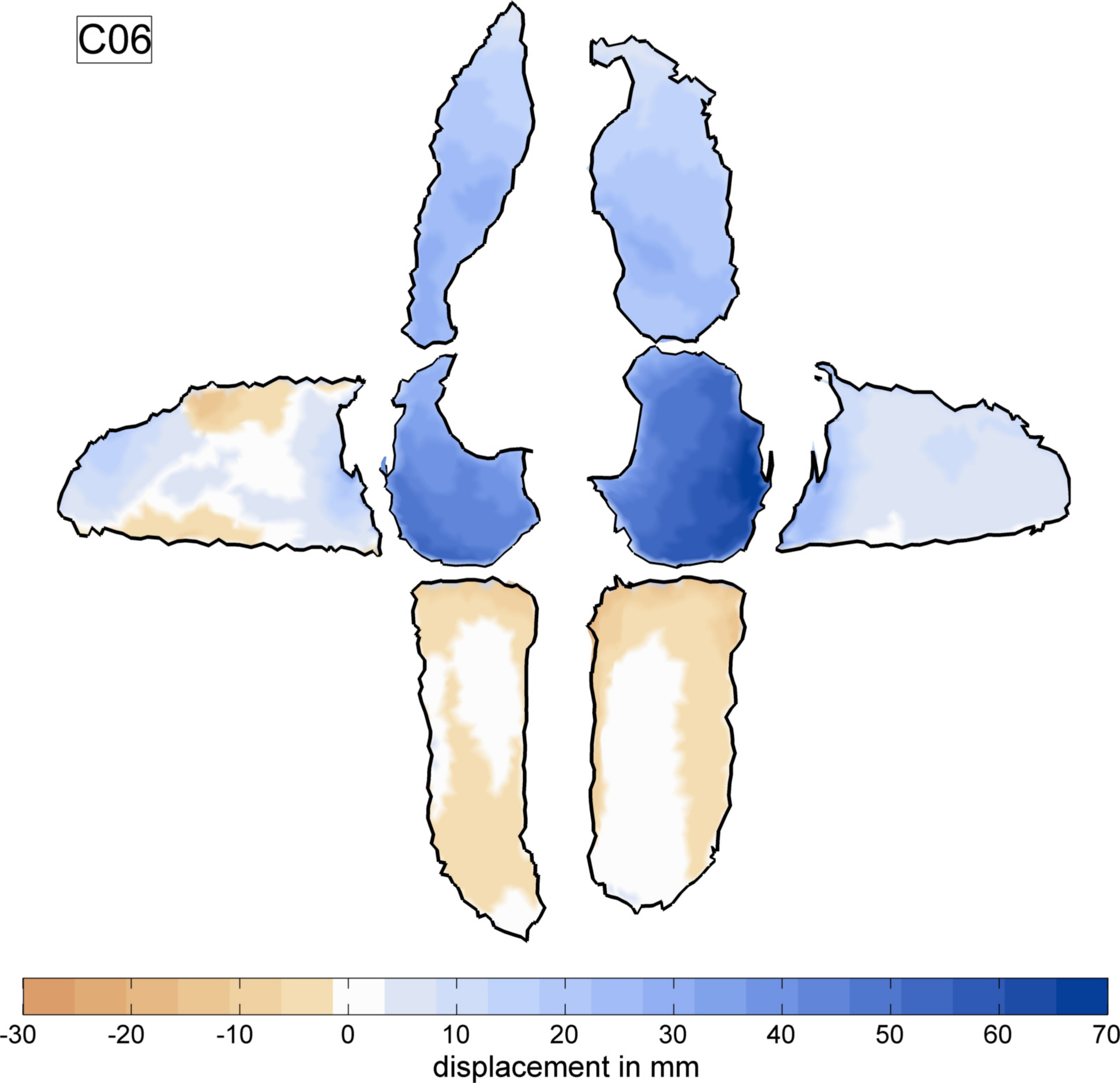
C06
-30
-20
-10
0
10
20
30
40
50
60
70
displacement in mm

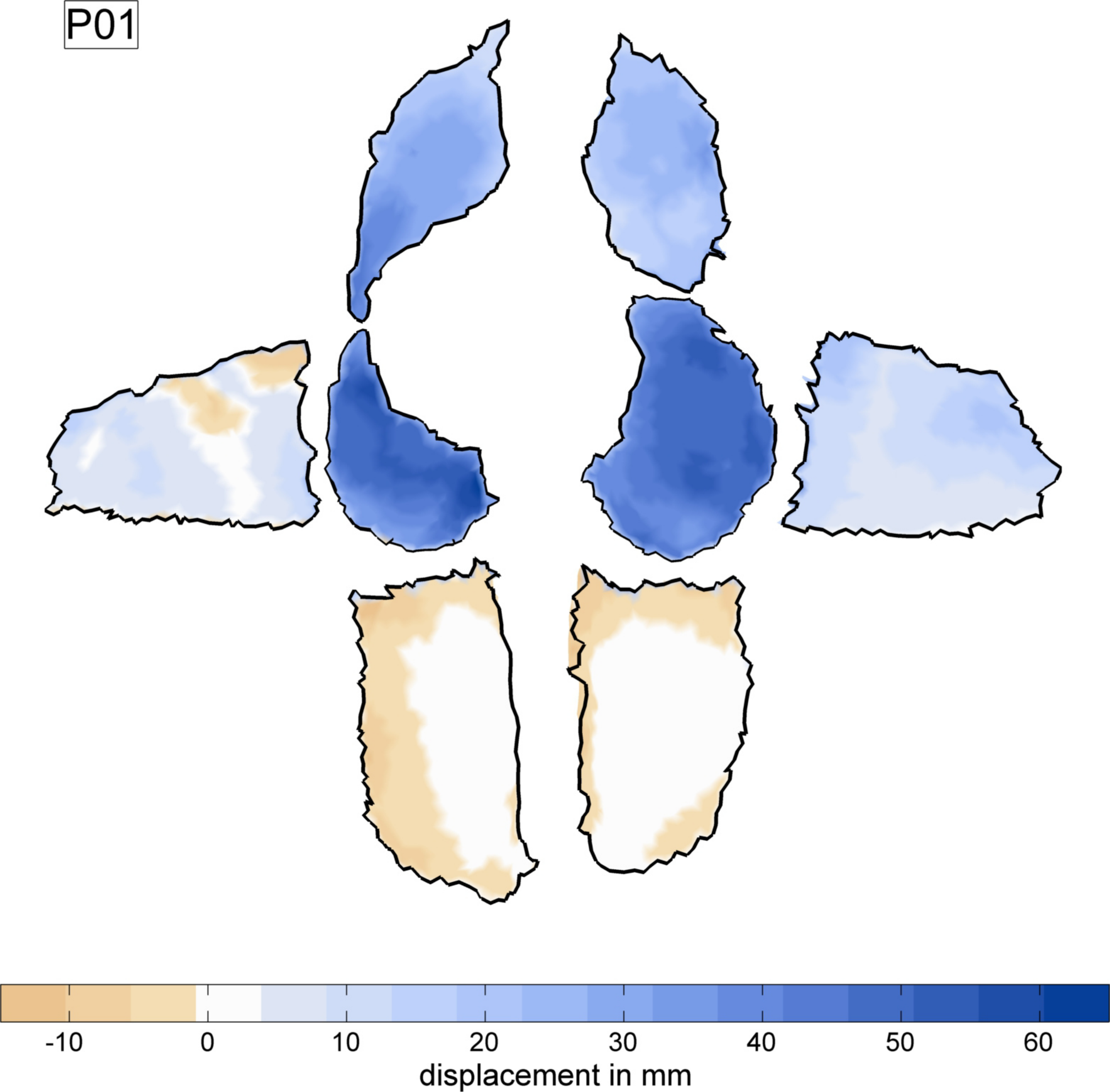
P01
-10
0
10
20
30
40
50
60
displacement in mm

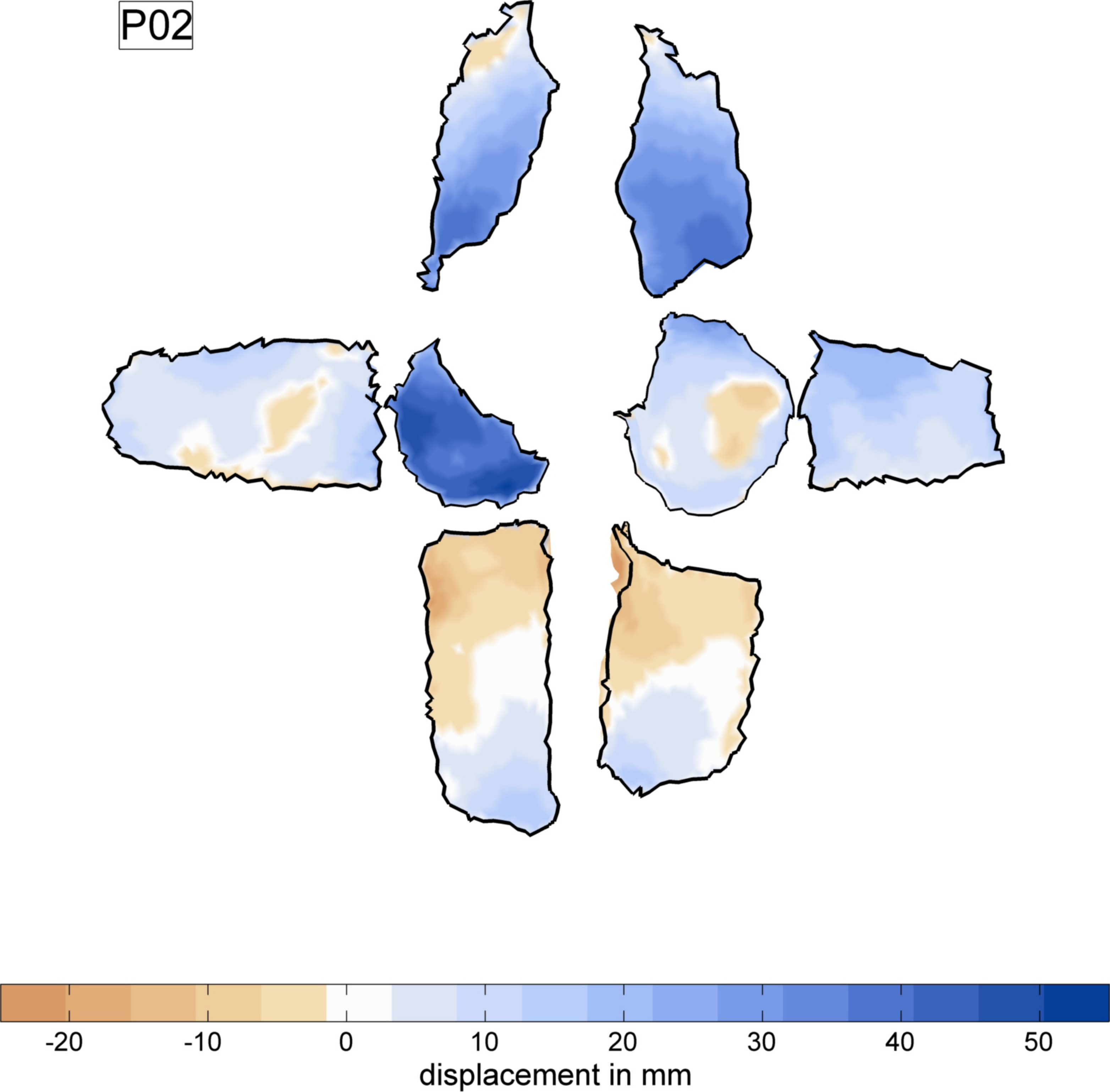
P02
-20
-10
0
10
20
30
40
50
displacement in mm

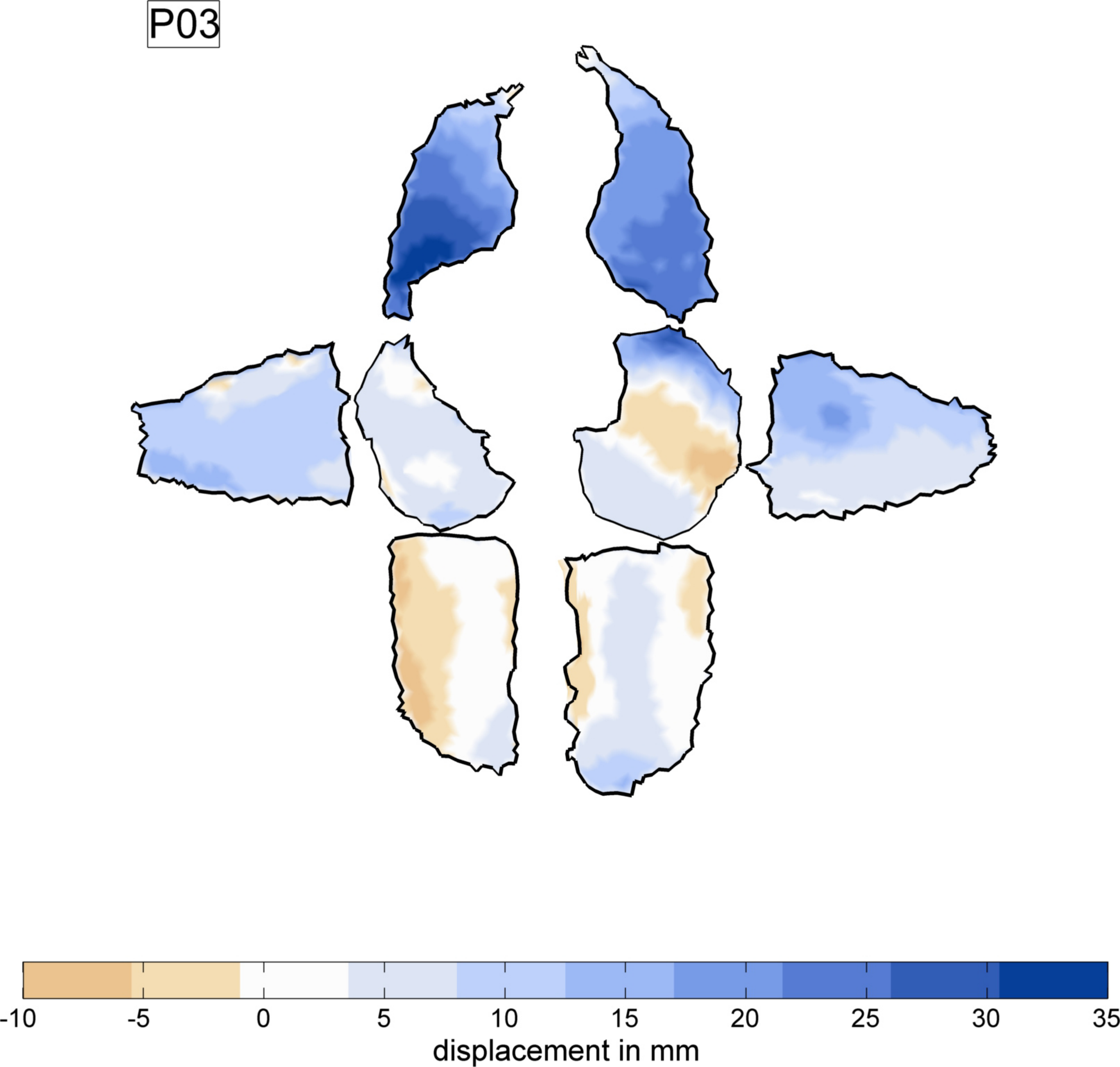
P03
-10
-5
0
5
10
15
20
25
30
35
displacement in mm

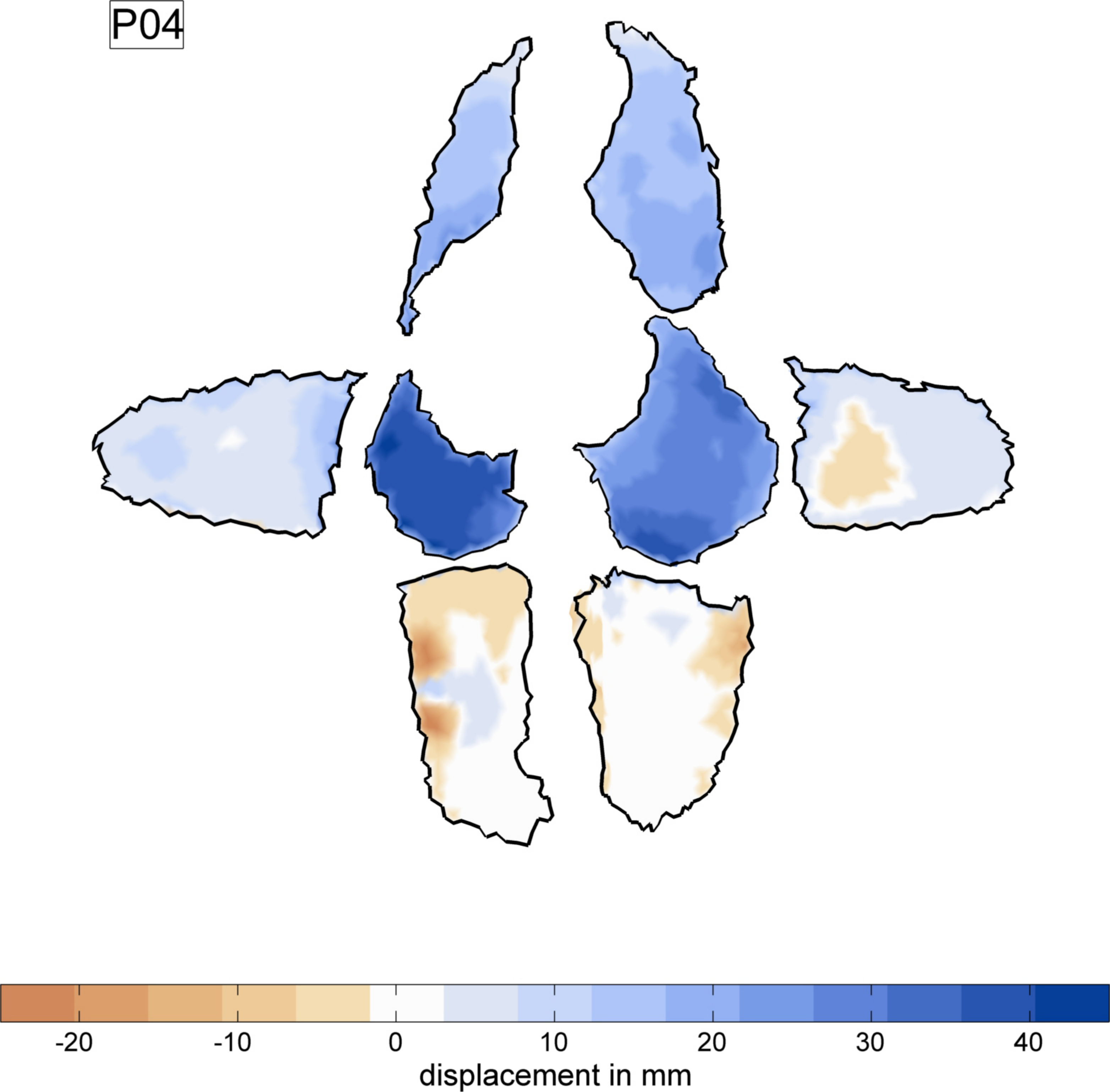
P04
-20
-10
0
10
20
30
40
displacement in mm

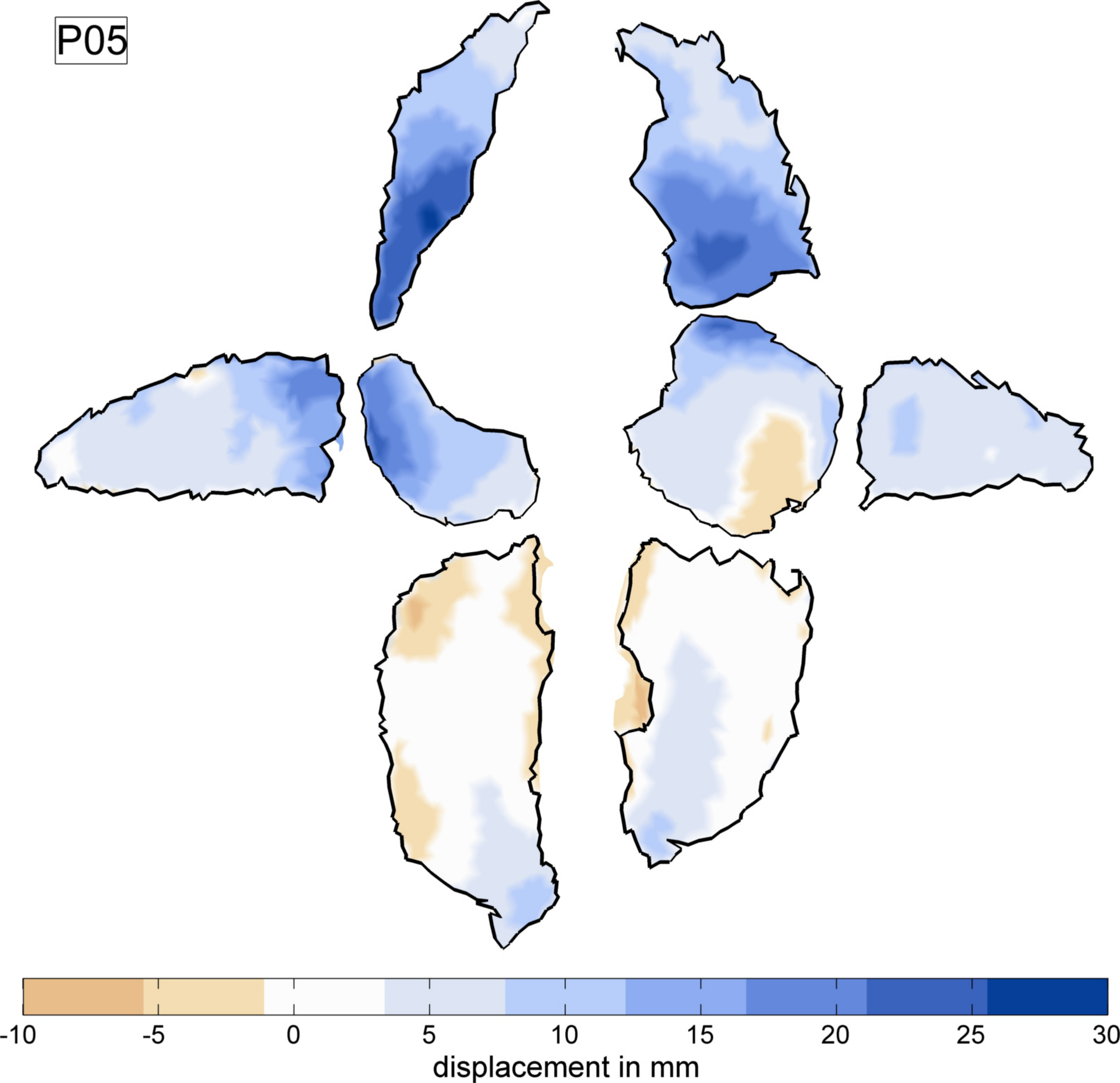
P05
-10
-5
0
5
10
15
20
25
30
displacement in mm

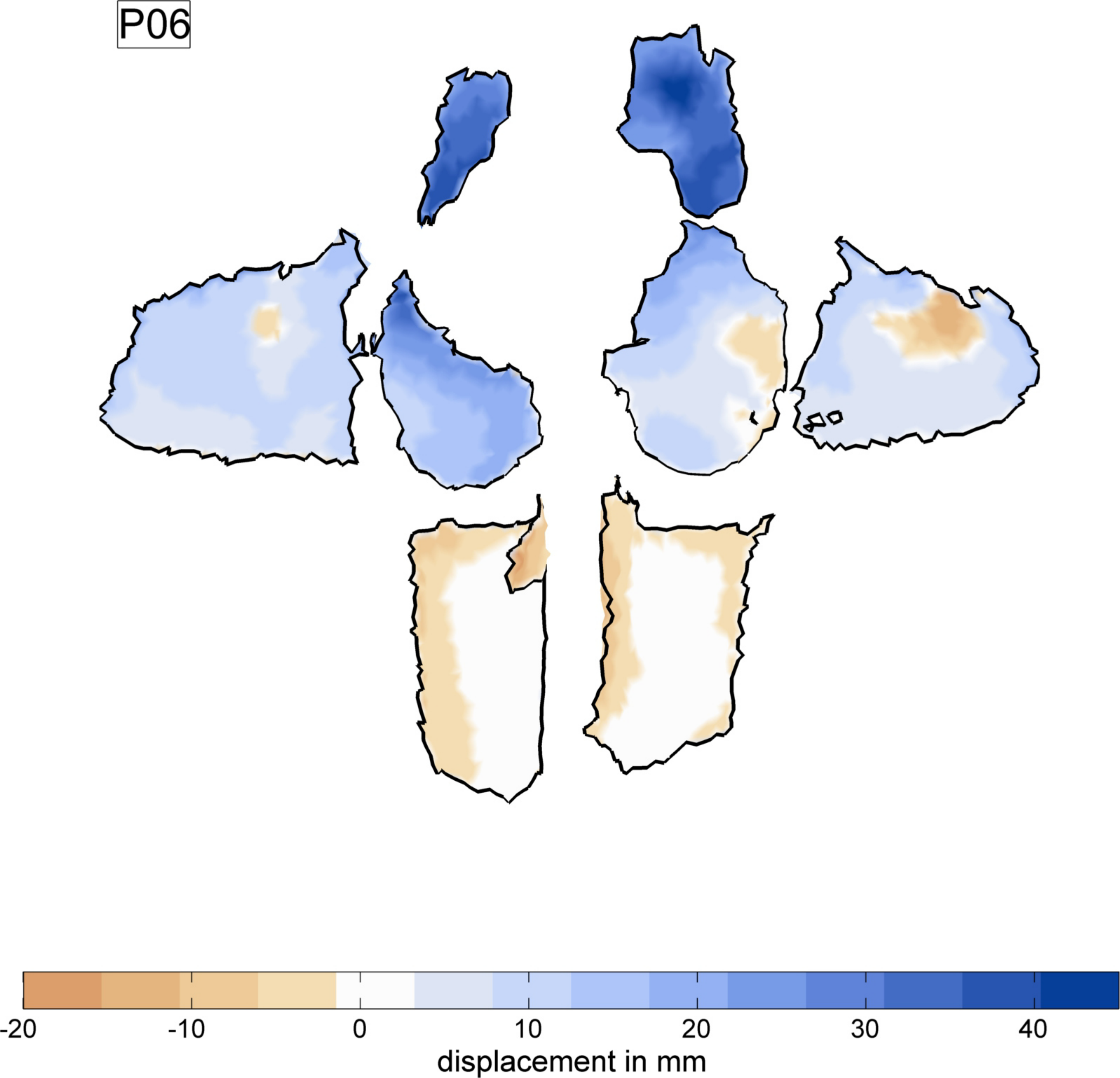

P06
-20
-10
0
10
20
30
40
displacement in mm

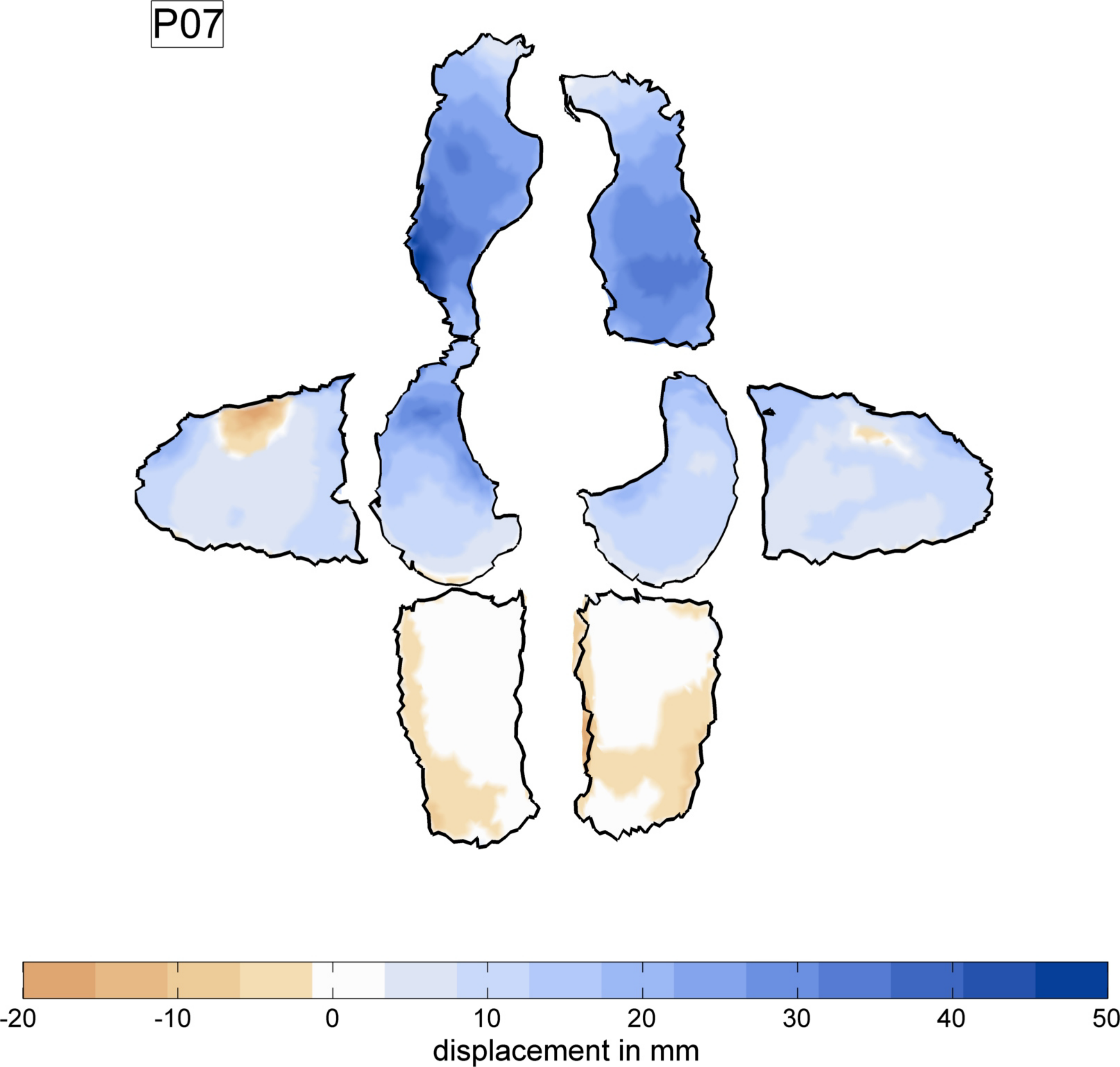
P07
-20
-10
0
10
20
30
40
50
displacement in mm

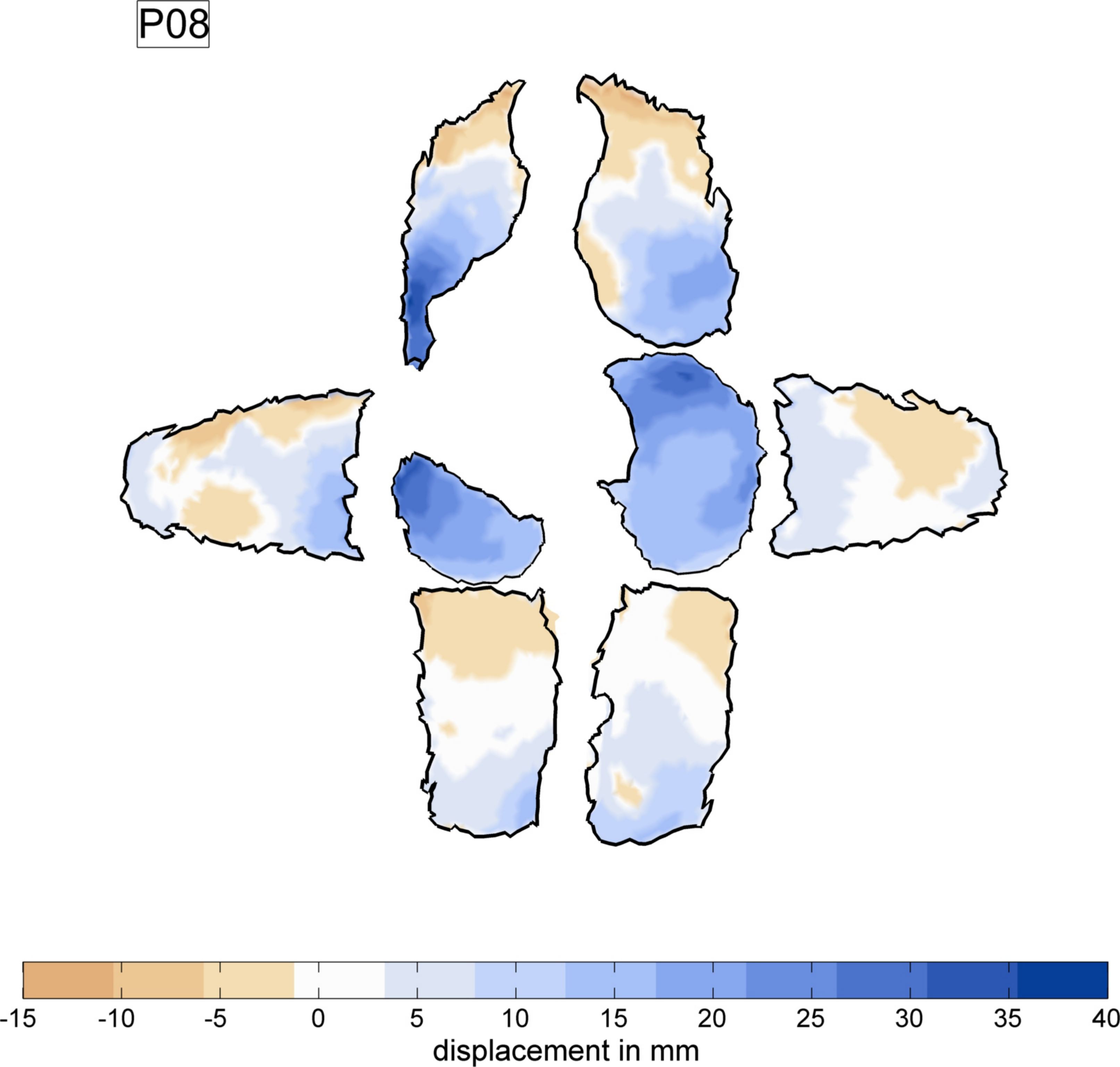
P08
-15
-10
-5
0
5
10
15
20
25
30
35
40
displacement in mm

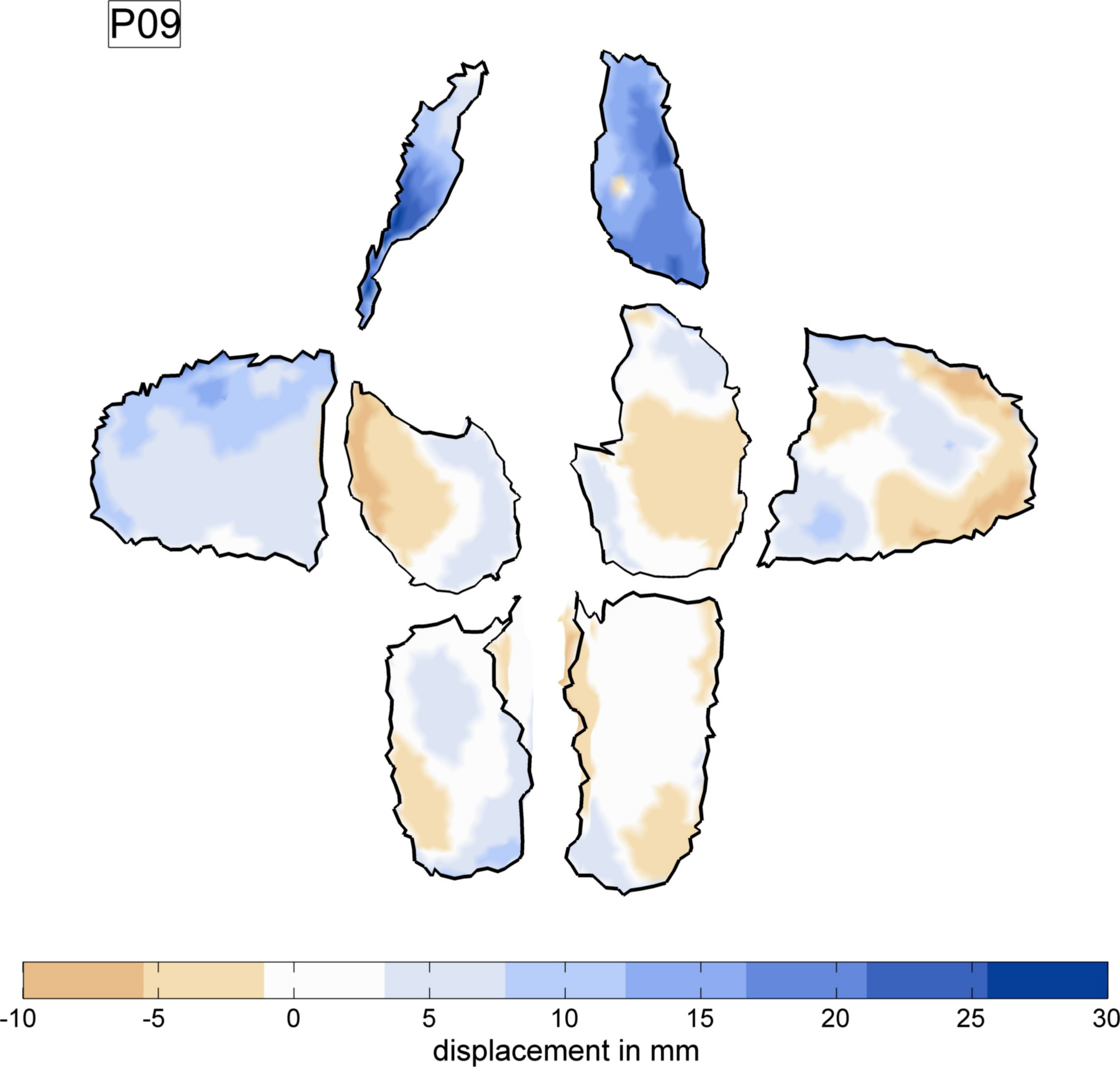
P09
-10
-5
0
5
10
15
20
25
30
displacement in mm

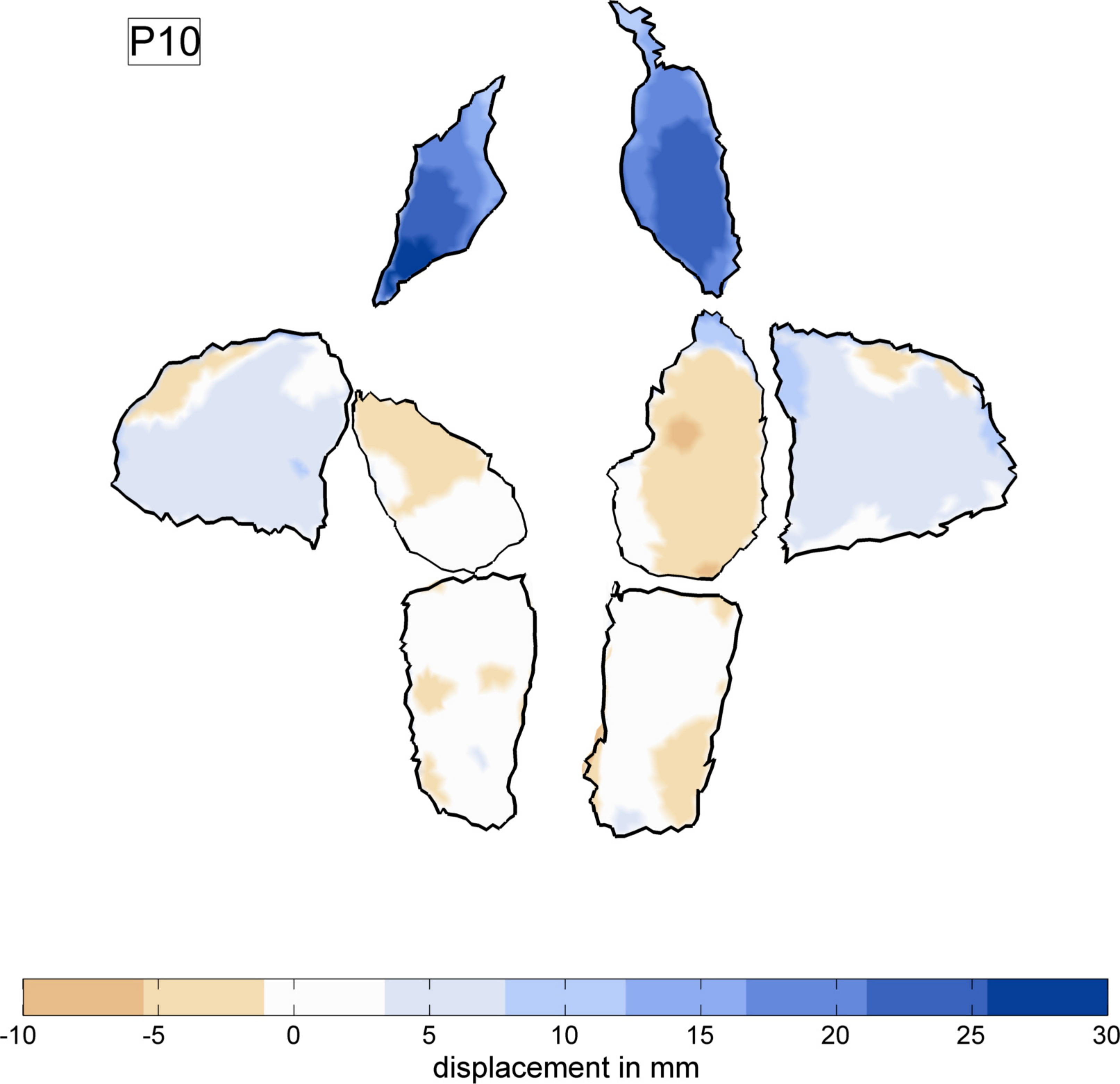

P10
-10
-5
0
5
10
15
20
25
30
displacement in mm
